# The myxozoan minicollagen gene repertoire was not simplified by the parasitic lifestyle: computational identification of a novel myxozoan minicollagen gene

**DOI:** 10.1186/s12864-021-07515-3

**Published:** 2021-03-20

**Authors:** Jiří Kyslík, Anush Kosakyan, Serafim Nenarokov, Astrid S. Holzer, Ivan Fiala

**Affiliations:** 1 Institute of Parasitology, Biology Centre, Academy of Sciences of the Czech Republic, Ceske Budejovice, Czech Republic; 2 Faculty of Science, University of South Bohemia, Ceske Budejovice, Czech Republic

**Keywords:** Myxozoa, Taxonomically restricted genes, Phylogeny, Cnidaria, Transcriptome, Custom made script

## Abstract

**Background:**

Lineage-specific gene expansions represent one of the driving forces in the evolutionary dynamics of unique phylum traits. Myxozoa, a cnidarian subphylum of obligate parasites, are evolutionarily altered and highly reduced organisms with a simple body plan including cnidarian-specific organelles and polar capsules (a type of nematocyst). Minicollagens, a group of structural proteins, are prominent constituents of nematocysts linking Myxozoa and Cnidaria. Despite recent advances in the identification of minicollagens in Myxozoa, the evolutionary history and diversity of minicollagens in Myxozoa and Cnidaria remain elusive.

**Results:**

We generated new transcriptomes of two myxozoan species using a novel pipeline for filtering of closely related contaminant species in RNA-seq data. Mining of our transcriptomes and published omics data confirmed the existence of myxozoan Ncol-4, reported only once previously, and revealed a novel noncanonical minicollagen, Ncol-5, which is exclusive to Myxozoa. Phylogenetic analyses support a close relationship between myxozoan Ncol-1–3 with minicollagens of *Polypodium hydriforme*, but suggest independent evolution in the case of the myxozoan minicollagens Ncol-4 and Ncol-5. Additional genome- and transcriptome-wide searches of cnidarian minicollagens expanded the dataset to better clarify the evolutionary trajectories of minicollagen.

**Conclusions:**

The development of a new approach for the handling of next-generation data contaminated by closely related species represents a useful tool for future applications beyond the field of myxozoan research. This data processing pipeline allowed us to expand the dataset and study the evolution and diversity of minicollagen genes in Myxozoa and Cnidaria. We identified a novel type of minicollagen in Myxozoa (Ncol-5). We suggest that the large number of minicollagen paralogs in some cnidarians is a result of several recent large gene multiplication events. We revealed close juxtaposition of minicollagens Ncol-1 and Ncol-4 in myxozoan genomes, suggesting their common evolutionary history. The unique gene structure of myxozoan Ncol-5 suggests a specific function in the myxozoan polar capsule or tubule. Despite the fact that myxozoans possess only one type of nematocyst, their gene repertoire is similar to those of other cnidarians.

**Supplementary Information:**

The online version contains supplementary material available at 10.1186/s12864-021-07515-3.

## Background

Myxozoa (Cnidaria) are multicellular parasitic organisms with very simple cellular organization. Their transition to parasitism from free-living cnidarians led to a distinct simplification of their body plan, which is reduced to parasitic plasmodial stages producing microscopic multicellular spores. Spores are typical stages of all myxozoans, serving as an agent of transmission between intermediate vertebrate hosts (fish) and definitive invertebrate hosts (annelids or bryozoans) [1]. Two different types of spores are developed during the myxozoan life cycle: actinospores in the invertebrate hosts and myxospores in the fish hosts. Myxozoan spores consist of shell valves enclosing an amoeboid sporoplasm containing an infectious germline. The process of host invasion is accompanied by the opening of the spore and release of the sporoplasm, which penetrates the host and develops intercellularly into multicellular plasmodial vegetative stages [2]. Thereafter, the plasmodium undergoes endogenous cell differentiation, leading to spore formation. Subsequently, the spores are released and serve as transmission stages.

The process of attachment of a myxozoan spore to the host is mediated by eversion of the polar tubules, which are coiled in highly complex organelles called polar capsules. These remarkable structures are homologs of cnidarian stinging organelles (nematocysts) and are the only synapomorphies that morphologically connect myxozoans and cnidarians. Although the marked similarity of myxozoan polar capsules and nematocysts was demonstrated more than a century ago [3–6], nematocyst-specific genes (minicollagens) were identified in the myxozoan genome only recently [7], confirming the classification of Myxozoa as cnidarians. Minicollagens are taxonomically restricted genes (TRGs) of cnidarians that are responsible for novel morphological structures. The importance of minicollagens is highlighted by their major role in the biogenesis of cnidarian nematocysts [8–12].

Minicollagens are a large family of proteins with a short central collagen triple-helical domain (12–16 Gly-X-Y repeats) flanked by variable polyproline stretches and N- and C-terminal cysteine-rich domains (CRDs). All minicollagen sequences have N-terminal signal peptides followed by a 4–20 residue propeptide sequence that is removed during capsule wall ontogenesis [13–15]. The minicollagens are a group of partially conserved genes that are expressed during the early phase of differentiation of cnidarian nematocysts. After nematocyte maturation, minicollagens are modified and anchored on the inner side of the wall in the form of highly compacted molecular structures [13, 16, 17]. Their multidomain motifs include small CRDs joined together by disulfide bonds and short central collagen sequences surrounded by polyproline repeats [18]. Hence, the different gene organizations and domain architectures of cnidarian minicollagens have given rise to 21 types of minicollagens identified in *Hydra*, which are classified into three derived groups [13, 19].

At least 30 different nematocyst types have been identified in cnidarians, with two to six different types in individual species [13, 20]. Myxozoan polar capsules are limited to only a single type, related to atrichous isorhizae [21].

The connection between myxozoan minicollagen and nematocyst diversity was suggested by loss of myxozoan minicollagen genes, with the initial identification of three Ncol types [19]. For comparison, *Polypodium hydriforme*, an enigmatic parasite of acipenserid eggs, and *Hydra* have 11 and 21 minicollagen types, respectively, and both species have a large number of nematocyst types (five in *Polypodium* and four in *Hydra*). Hence, the myxozoan minicollagen repertoire was presumed to be dramatically reduced [19]. Conversely, the discovery of the fourth myxozoan minicollagen type suggested a higher polar capsule gene complexity [22]. Nevertheless, the disparity in protein contents assumed an alternative explanation with high homogeneity of the polar capsule [23].

Myxozoan polar capsules are morphologically complex subcellular organelles with a more limited molecular structure than the nematocysts of their free-living cnidarian relatives [21]. The proteomic composition of the nematocyst consists of a cluster of proteins unique to Cnidaria [24, 25]. A substantial part (18%) of the *Hydra* nematocyst proteome comprises specialized extracellular matrix components along with a group of structural proteins, the minicollagens [24].

Reconstruction of the evolutionary history of minicollagens is limited by the structural constraints on sequence variation, as well as by extensive repetitive elements. However, phylogenetic analysis of minicollagen genes supports the close relationship between Myxozoa and *P. hydriforme*, a cnidarian parasite of sturgeons [19]. This close relationship was previously indicated by several studies based on a universal marker, SSU rDNA, as well as by multigene analyses [7, 26–30]. The affinity of *P. hydriforme* and Myxozoa was also revealed by the close relationship between a novel myxozoan type of minicollagen (Ncol-4) identified by bioinformatic analysis of *Myxobolus pendula* transcriptome and *P. hydriforme* minicollagens Ncol-7, Ncol-8, and Ncol-9 [22].

We selected two myxozoan species, *Myxidium lieberkuehni* and *Nephrocystidium pickii*, for transcriptomic analysis and mining of minicollagen genes. *M*. *lieberkuehni* Bütschli, 1882 infects the urinary bladder of northern pike *Esox lucius* L. (Actinopterygii: Esocidae) and has plasmodial stages with developing spores that usually occur in heavy infections and with high prevalence [31]. *M*. *lieberkuehni* co-occurs with *N. pickii* Weißenberg, 1921, which was identified in the renal corpuscles of pike kidneys; the parasite causes hypertrophy of host cells (xenomas) filled with a large number of parasitic cells [32]. *N. pickii* was considered as an extrasporogonic proliferative stage of *M*. *lieberkuehni* [31, 33, 34]. Re-investigation of *N*. *pickii* by phylogenetic analysis supported this species as a different taxon, albeit phylogenetically very closely related, with an unknown sporogonic stage [35]. This is an example of the common phenomenon of parasitic co-infections in the same host. Such co-infections are often problematic for molecular-level species determinations using phylogenetic markers. Co-infections are even more problematic for transcriptome or genome assembly pipelines. Besides the development of various tools to alleviate or remove such contamination from RNA-seq data [36, 37], some inherent limits remain, including filtering of very closely related species [38].

In the present study, using a well-established custom-made de novo script for filtering of closely related species, we generated transcriptomes of two closely related myxozoan parasites co-infecting the same fish host. In addition, through bioinformatic analyses of available omics data, we identified a new myxozoan minicollagen, Ncol-5. We described the molecular structure of this new protein and its phylogenetic relationship to known cnidarian and myxozoan minicollagens. Our bioinformatic analysis revealed myxozoan Ncol-4 minicollagen in eight species, confirming its existence in Myxozoa. Thorough mining of myxozoan and cnidarian genomes and transcriptomes enabled us to identify various new minicollagen paralogs in myxozoan and cnidarian species, and thus to better reconstruct the evolutionary history of cnidarian minicollagens.

## Results

### Transcriptome assemblies

**Fig. 1:**
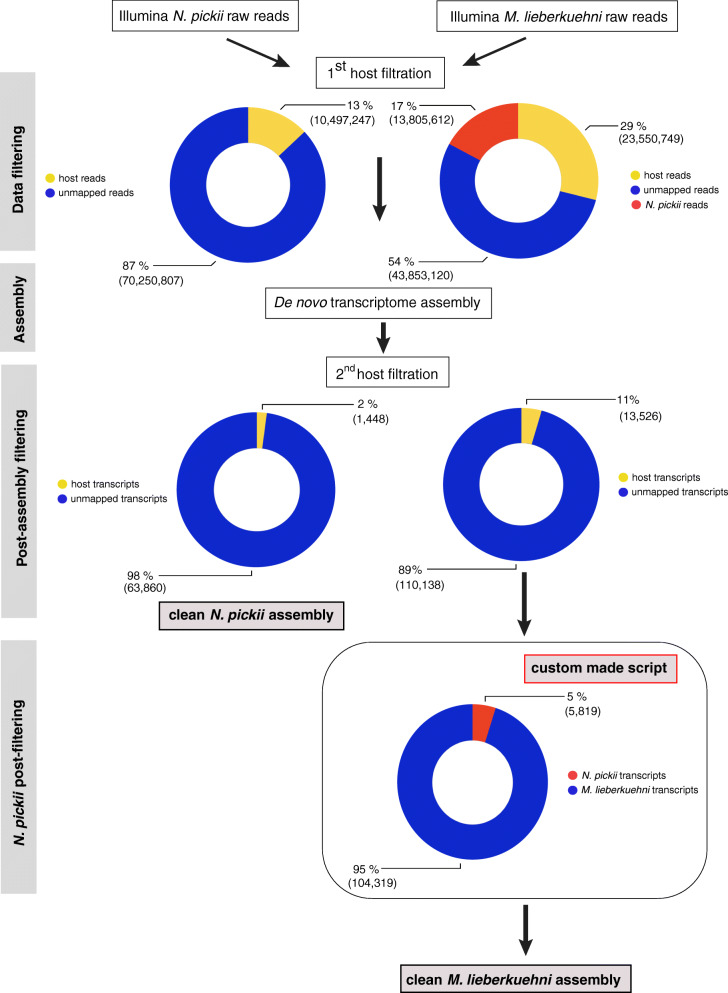
Workflow for de novo transcriptome assembly and analysis pipeline to filter out coinfection by a closely related species, thus generating clean data

RNAs isolated from *M. lieberkuehni* and *N. pickii* infecting the excretory system of common pike *E. lucius* were subjected to high-throughput sequencing on the Illumina HiSeq™ 2000 platform, yielding a total of 81,209,482 and 80,748,054 paired 100 bp reads, respectively. A schematic representation of the de novo transcriptome reconstruction pipeline is shown in Fig. 1. Raw reads of both transcriptomes isolated from the urinary bladder and kidney of pike revealed host contamination, and the only transcriptome of the sporogonic stages of *M*. *lieberkuehni* from urinary bladder was contaminated by *N*. *pickii*; we detected the presence of the SSU rDNA gene of *N. pickii* in the *M. lieberkuehni* transcriptome. Overall, 28.8% *M. lieberkuehni* and 12.8% *N. pickii* raw reads were mapped to the host (*E. lucius* genome, Acc. No. GCA_011004845.1) and filtered out from the transcriptome. The remaining unmapped reads of *N. pickii* transcriptome were assembled, resulting in 65,308 transcripts with N50 = 4003 bp. After post-assembly filtration, 1448 transcripts of *N*. *pickii* assembly appeared to have host hits and were removed from the assembly, resulting in a total of 63,860 transcripts with N50 = 3974 bp and GC = 31.12%.

In addition, after host filtration, 17% of *M. lieberkuehni* raw reads were mapped to the *N. pickii* assembled transcriptome. The remaining unmapped reads of the *M. lieberkuehni* transcriptome were assembled, resulting in 123,664 transcripts with N50 = 1341 bp. After post-assembly filtration, 13,526 transcripts of *M. lieberkuehni* assembly appeared to have host hits and were removed from the assembly, resulting in a total of 110,138 transcripts with N50 = 1343 bp and GC = 32.08%. Post-assembly matching to the *N. pickii* transcriptome using a novel custom-made hit-coverage filtering script revealed 33,873 transcripts containing any hit to the contaminant species, of which 5819 were more than 90% similar to *N. pickii*. These 5819 contaminated transcripts were eliminated using a novel hit-coverage filtering script, resulting in a final clean assembly of 104,319 valid transcripts from the original *M*. *lieberkuehni* transcriptome with N50 = 1351 bp and GC = 31.90%.

### Ncol-5, a novel myxozoan minicollagen

**Fig. 2:**
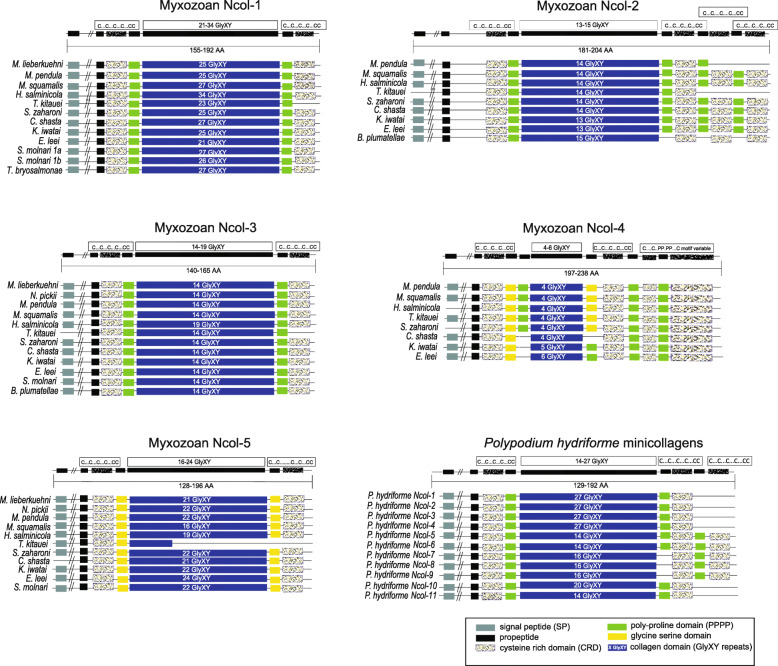
Schematic drawings of myxozoan and *Polypodium hydriforme* minicollagens. See legend for domain structure, shown in colored boxes. Ncol-5 of *Thelohanellus kitauei* represents a partial sequence

A BLAST search focused on identifying minicollagen homologs in the newly generated transcriptomes of *M. lieberkuehni* and *N. pickii* revealed a novel myxozoan minicollagen type that we named Ncol-5. Using Ncol-5 as a query, we successfully identified a homolog of this minicollagen in all other nine myxozoan species with available genomic and transcriptomic data (Additional file 1). BLAST searches of other cnidarians failed to identify any homolog of myxozoan Ncol-5. The Ncol-5 protein sequence consists of 177 amino acids in *M. lieberkuehni* and 179 in *N. pickii* sp., including signal peptide, a propeptide region missing the conserved KR dipeptide motif, canonical N-terminal CRD (C...C...C...C...CC), and noncanonical C-terminal CRD motifs (C...C……...C...C...CC). Ncol-5 homologs in all 11 myxosporeans possess 16–24 Gly-XY repeats in their central collagen triple-helix domain (Fig. 2). The novel minicollagen contains a specific amino acid composition of predominant serine and glycine repeats surrounding the collagen domain and replacing the Poly-P domains present in other minicollagens (Fig. 2, Additional file 2). Our BLAST searches did not identify Ncol-5 in EST data from the malacosporean *Buddenbrockia plumatellae* or *Tetracapsuloides bryosalmonae*.

PCR amplification of Ncol-5 revealed a single intron positioned in the propeptide region with a length of 32 and 40 bp in *M*. *lieberkuehni* and *N. pickii*, respectively (Additional file 3). PCR also supported the absence of the conserved terminal KR dipeptide motif in the propeptide sequence.

Three-dimensional (3D) structural homology analyses of the N-terminal CRD of newly identified Ncol-5 of *M. lieberkuehni* confirmed the homology to known nuclear magnetic resonance (NMR) structures of the N-terminal domain (CRD) of *Hydra vulgaris* Ncol-1 (Additional file 4). Protein homology searches of N-CRD of *M*. *lieberkuehni* Ncol-5 revealed a 93% structure probability and 61% sequence identities with the NMR structure of *H. vulgaris* Ncol-1 (1ZPX). The 3D structure of canonical N-CRD of Ncol-5 is highly similar to the published structure Ncol-1 of *H. vulgaris*, as supported by accuracy testing of the superimposed structures.

### Myxozoan minicollagen Ncol-4

**Fig. 3:**
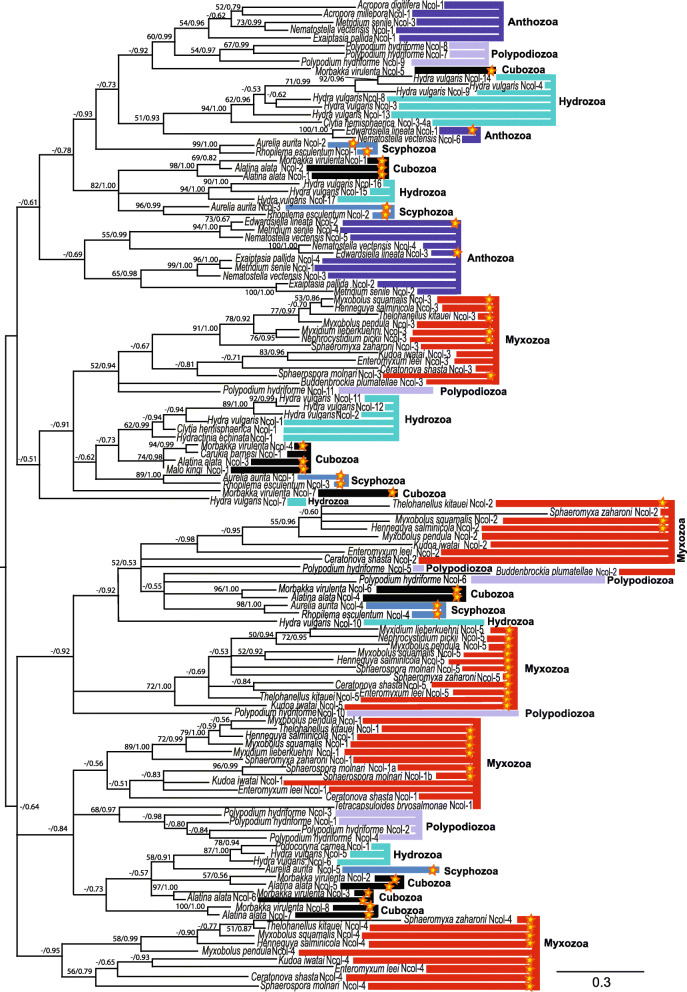
Bayesian inference analysis of cnidarian minicollagen paralogs. Additional file 2 contains detailed information on each analyzed sequence. Newly obtained sequences are highlighted by stars. Nodal support is shown for maximum likelihood (ML) bootstraps and Bayesian inference (BI) posterior probability. The scale bar is provided under the tree

Using the only known Ncol-4 from *M. pendula* as a query, we successfully mined Ncol-4 minicollagen homologs from genomes/transcriptomes of eight myxosporean species (Additional file 1). All sequences exhibited canonical minicollagen features with a signal peptide, a propeptide region, cysteine-rich domains, central Gly-X-Y domain, and a conserved N-terminal CRD (C...C...C...C...CC) with a unique C-terminal CRD. Ncol-4 was not identified in the newly generated transcriptomes of *M. lieberkuehni* and *N. pickii* from pike, from the transcriptome of blood stages of *Sphaerospora molnari*, or from malacosporean species. A larger sampling of myxozoan Ncol-4 sequences helped to better clarify the structure of myxozoan Ncol-4. The identified Ncol-4 sequences revealed high sequence similarity with the first described Ncol-4 of *M. pendula* [22]. Ncol-4 has a shortened Gly-X-Y tripeptide domain, with no polyproline following Gly-X-Y, and a unique C-terminal CRD domain interrupted by a polyproline stretch. In six myxosporeans (including *M. pendula*), the tripeptide domain contains only four repeats of Gly-X-Y rather than seven, as documented in [22]. Lack of alanine in the fifth repeat of the Gly-X-Y, is replaced by the glycine residue [22] in a modified polyproline domain. The myxosporeans *Kudoa iwatai* and *Enteromyxum leei* have five and six Gly-X-Y repeats, respectively, with proline residues following the Gly-X-Y domain. Like myxozoan Ncol-5, Ncol-4 has an atypical C-terminal polyproline domain character, including predominant glycine and serine residues. Moreover, the *E. leei* N-terminal polyproline domain lost all proline residues and mostly consists of glycine and serine. Interestingly, *P. hydriforme* Ncol-7–9 have only two (non-proline) amino acid residues between Gly-X-Y and C-terminal CRD. This peculiarity suggested close relationships between *P. hydriforme* Ncol-7–9 and myxozoan Ncol-4, but our analysis did not support this scenario; by contrast with [22], phylogenetic reconstruction indicated separated clustering instead (Fig. 3). Furthermore, the analysis also associated the Ncol-2–like minicollagen found in the *Ceratonova shasta* proteome [39] with a myxozoan Ncol-4 cluster.

### Myxozoan genomic architecture of minicollagens

**Fig. 4:**
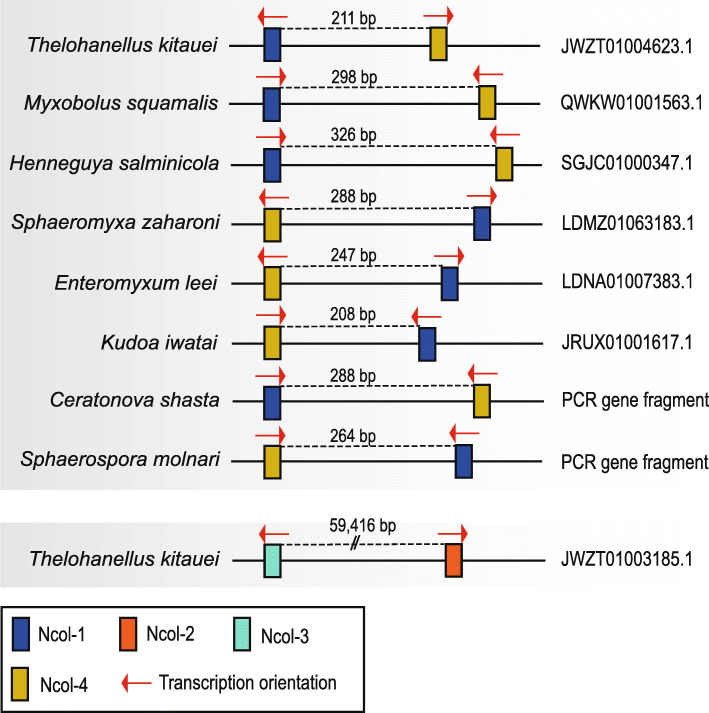
Schematic illustration of the myxozoan minicollagen position in the genome

In the available genomes of Myxozoa, we identified genomic regions containing clusters of minicollagens (Fig. 4) in a total of six myxozoan species: *Thelohanellus kitauei*, *H. salminicola*, *M. squamalis*, *K. iwatai*, *S. zaharoni*, and *E. leei*. The genomic positions of Ncol-1 and Ncol-4 of *S. molnari* and *C. shasta* were determined by PCR amplification of total DNA. Ncol-1 and Ncol-4 have similar gene arrangements with a short variable noncoding intergenic region (IGR) (208–326 bp). Interestingly, the Ncol-1 and Ncol-4 gene clusters exhibited an incongruence between the species in the direction of transcription and gene order. Additionally, in the genome of *T. kitauei*, we identified an additional cluster of Ncol-2 and Ncol-3 with a relatively long IGR (59,416 bp), whereas Ncol-2 and Ncol-3 of other myxozoan species are not colocalized on one scaffold. Close clustering of Ncol-5 with other myxozoan minicollagens was not detected in any species studied.

### Evolution of myxozoan and cnidarian minicollagens

The phylogenetic tree showed all newly identified myxosporean Ncol-5 sequences clustering in a single, clearly distinct clade with relatively high nodal support (Fig. 3). The closest minicollagen homologs to myxozoan Ncol-5 were *P. hydriforme* Ncol-10 and a group of minicollagens including *H. vulgaris* Ncol-10, two representatives each from Cubozoa and Scyphozoa, and a set of myxozoan Ncol-2 minicollagens closely related to *P. hydriforme* Ncol-5 and Ncol-6. Besides newly determined myxozoan minicollagens, we identified several cnidarian minicollagen genes by mining of available cnidarian genomes and transcriptomes.

Cnidarian evolution of minicollagen genes (Fig. 3) includes five lineages of myxozoan minicollagens (Ncol-1 to Ncol-5). Their close relationships to the cnidarian homologs are difficult to assess due to large polytomies resulting from Bayesian inference (BI) analysis (Fig. 3) and low bootstrap support of relatively well-resolved maximum likelihood (ML) analysis (Additional file 5). ML analyses revealed that three out of five myxozoan minicollagens had phylogenetic affinity with minicollagens of *P. hydriforme* (Additional file 5): myxozoan Ncol-1 with a group of multiplicated *P. hydriforme* minicollagens, Ncol-1 to Ncol-4; myxozoan Ncol-2 with *P. hydriforme* Ncol-5 and Ncol-6; and myxozoan Ncol-3 with *P. hydriforme* Ncol-11. BI analysis supported only the sister relationship of myxozoan Ncol-3 and *P. hydriforme* Ncol-11. The other phylogenetic relationships mentioned above were not resolved in BI and are displayed as large polytomies with other cnidarian minicollagens. Minicollagen Ncol-4 and Ncol-5 do not have any close relatives, instead representing independent lineages within cnidarian minicollagens.

Phylogenetic reconstruction of the cnidarian minicollagens suffers from very low nodal supports and unstable topologies at lower (i.e., more ancient) nodes of the phylogenetic tree (Fig. 3). Lack of suitable outgroup sequences prevents us from rooting the tree with an outgroup sequence; consequently, we cannot deduce ancestral minicollagen lineages. The evolution of cnidarian minicollagens is characterized by the formation of clusters of minicollagens from the same cnidarian taxonomic group. We can recognize three anthozoan clusters. One of these clusters contains triplication of *Nematostella vectensis* and *Metridium senile* and duplication of *Edwardsiella lineata* and *Exaiptasia pallida*. Medusozoa, Scyphozoa, Cubozoa, and Myxozoa form five phylogenetically distinct clusters of minicollagens, and Hydrozoa and Polypodiozoa form six. *H. vulgaris* has a large number of minicollagen paralogs as a result of two large multiplication events, one resulting in six paralogs (Ncol-3, 4, 8, 9, 13, and 14) and the other in four (Ncol-1, 2, 11, and 12). Multiplication is also typical for *P. hydriforme* minicollagens. By contrast, we observed no multiplications in Scyphozoa and only one in Myxozoa (*S. molnari* Ncol-1a and Ncol-1b).

## Discussion

Our study revealed that the myxozoan repertoire of minicollagen genes is less reduced, as previously indicated [19]. Our mining of myxozoan transcriptomes and genomes supported the existence of Ncol-4 by revealing seven new myxosporean Ncol-4 sequences with high sequence similarity and close phylogenetic relationships to *M. pendula* Ncol-4 [22]. We also revealed the presence of a fifth myxozoan minicollagen, Ncol-5, which we identified in 11 myxosporean species. Myxozoan Ncol-5 is structurally similar to myxozoan Ncol-1 and Ncol-3 in terms of the size of the central Gly-X-Y (collagen) domain region, which is surrounded by single CRDs at both ends. Ncol-5 can be classified as a group 1 minicollagen based on CRD similarity [13]. Both Ncol-5 domains are distinctive from other myxozoan minicollagens. Ncol-5 lacks proline-rich-domains but has regions rich in serine and glycine (Additional file 4). Similar modifications of the polyproline domain can also be found in other cnidarians. Cubozoans *Morbakka virulenta* Ncol-7 and *Alatina moseri* Ncol-2 have modifications in the first polyproline region (between the CRD and Gly-X-Y domain) characterized by a glycine-rich region followed by alanine-proline repeats. *Hydra vulgaris* Ncol-15–17 contain a relatively long region with a reduced number of proline amino acids and several serines and glycines. More importantly, five minicollagens of *P. hydriforme*, Ncol-7–11, contain polyproline regions that are noncanonical, with a small number of prolines and an abundance of glycine, serine, and alanines (Additional file 2).

Reconstruction of the evolutionary history and phylogenetic relationships of the minicollagen gene family is challenging. The overall structural constraints and repetitive sequence elements of minicollagens prevent confident phylogenetic inference [13]. Given the ambiguity in alignment construction, we decided to exclude the polyproline domains from the dataset, even in closely related species. Nonetheless, we observed a high degree of instability in the phylogenetic trees as a consequence of the low phylogenetic signal of these protein-coding genes. The inconsistencies in evolutionary tree topologies correlated with the applied method reflected the difficulty of reliably revealing the evolution of cnidarian minicollagens.

Our analysis supports the previously reported relationship between three myxozoan minicollagens (Ncol-1, 2, and 3) and minicollagens of *P. hydriforme* [19, 22]. Despite the polytomic character of minicollagen gene evolution, BI analysis revealed a more conservative picture of all myxozoan minicollagens. ML analysis supported a close relationship between myxozoan minicollagens Ncol-1–3 and *P. hydriforme*. Nonetheless, myxozoan Ncol-4 lacks affinity for *P. hydriforme*, as shown previously [22]. Our BI analysis also revealed a close relationship between myxozoan Ncol-5 and *Polypodium* Ncol-10, which strengthens the idea of a common evolutionary history of the Myxozoa and *P. hydriforme* [30].

We were unable to identify myxozoan Ncol-4 and Ncol-5 in malacosporeans, myxozoans with soft-wall malacospores, or parasitism in bryozoan hosts, suggesting that these two minicollagens are unique only for myxosporeans. Ncol-4 and Ncol-5 may have evolved after the myxosporean–malacosporean split from an ancestral minicollagen ortholog, or malacosporeans may have lost these genes over the course of evolution. The absence of Ncol-4 and Ncol-5 in malacosporeans may also be explained by the available data (March 2020), as ESTs may not include all minicollagens present in the genome of *B. plumatellae* and *T. bryosalmonae*.

We conclude that the myxozoan minicollagen repertoire is similar to that in other cnidarian groups but includes two types unique to myxozoans, more specifically myxosporeans (Ncol-4 and Ncol-5). Our analysis revealed five clusters of minicollagen homologs in Myxozoa, as well as in Scyphozoa and Cubozoa; six clusters in Hydrozoa and *Polypodium*; and only three clusters in Anthozoa. The evolutionary reconstruction indicated that the 21 known minicollagen types reported in *Hydra* [24] are a result of recent evolutionary multiplication of ancestral types of the minicollagen, in various clusters. Consequently, the number of main minicollagen clusters is similar in all medusozoan groups, including parasitic Myxozoa. This multiplication and the resultant variety of minicollagen homologs in *Hydra* and other cnidarians are consistent with the hypothesis that the diversity of minicollagen is linked to nematocyst diversity, which is highest in Hydrozoa [13]. Only a single nematocyst type has been reported in Myxozoa, and this may explain the limited multiplication of the minicollagen types within the myxozoan minicollagen gene repertoire (only one duplication in *S*. *molnari*).

Our analysis does not include polyproline domains in the alignment, as we found them too variable to be unambiguously aligned. However, when we kept these regions and performed the phylogenetic analysis, neither ML nor BI supported a close relationship among these homologs (data not shown). Therefore, we speculate that wider taxon sampling of myxozoan Ncol-4 may influence its phylogenetic position. Myxozoan Ncol-4 represents a rather independent minicollagen lineage with no closely related cnidarian taxa. *P. hydriforme* Ncol-7–9 clustered in the large group of minicollagens representing all cnidarian taxa, including all three anthozoan clusters of minicollagen homologs. We can expect the existence of one more myxozoan minicollagen type that would cluster within the aforementioned group of *P. hydriforme* minicollagens. Alternatively, the absence of this missing myxozoan minicollagen may be the result of gene loss over the course of evolution of the parasitic lifestyle of myxozoans.

Minicollagen genes are organized into clusters in the genome, with collinear expression in representatives of Anthozoa, Hydrozoa, Cubozoa, and Scyphozoa [10, 13, 40]. We identified myxozoan Ncol-1 and Ncol-4 on a single contig in very close proximity, supporting a similar gene organization in Myxozoa (Fig. 4). The close physical proximity of Ncol-1 and Ncol-4 at the genome level suggests an ancestral gene duplication event and subsequent neofunctionalization of either Ncol-1 or Ncol-4. However, the other three myxozoan minicollagens are unlikely to be organized in clusters, as indicated by the long distance between Ncol-2 and Ncol-3 in the genome of *T. kitauei*. Interestingly, we identified rearrangement of gene order in the Ncol-1/Ncol-4 gene cluster as well as different transcription directions, potentially suggesting diverse genomic organization and noncollinear expression relative to cnidarian minicollagen gene clusters [40].

Our newly generated transcriptome of *N. pickii* provides novel transcriptomic data of myxozoan extrasporogonial stages during which extensive proliferation of the parasite is typical, and spore formation is never observed [31, 35]. Moreover, these stages of *N. pickii* fill the cytoplasm of endothelial cells of the host glomerular capillary and thus represent an atypical myxozoan xenoma-like intracellular development stage. The detection of minicollagen transcripts in these extrasporogonic myxozoan stages was surprising. We detected two minicollagen genes, Ncol-3 and Ncol-5, in the transcriptome of the xenoma stage of *N. pickii*, as well as Ncol-1, 3, and 5 in the transcriptome of blood stages of *S. molnari*. We assume that the future estimated expression levels of these minicollagens might be very low in these stages and increase in later spore-forming stages. However, myxozoan Ncol-3 and Ncol-5 might be the minicollagens expressed earliest in myxozoan development, with basal levels of transcription detectable in the non-spore forming stages. Sequence differences in the Ncol-3 and Ncol-5 of *N*. *pickii* and *M. lieberkuehni* supported the SSU rDNA-based analysis in [35], which proved the nonconspecificity of the pike parasites *M*. *lieberkuehni* and *N*. *pickii*.

The lack of proline residues, together with a large number of glycine and serine residues between the CRDs and the tripeptide domain documented in Ncol-5 and to a lesser extent in Ncol-4, may represent an adaptation of myxozoan minicollagen genes to some specific function of these parasitic cnidarians. Ben-David et al. [41] reported a contraction of the myxozoan polar tubule after its release from the myxozoan polar capsule, causing the spore to move closer to the host surface for entry of the infective sporoplasm. Such polar tubule elasticity might be related to a unique myxozoan feature not present in nematocyst tubes of free-living cnidarians [41]. The glycine/glutamine-rich domain is part of the elastic protein Cnidoin, which was discovered in the proteome of *Hydra* nematocysts [24]; its elastic sequence is homologous to the glycine-rich region of the spider silk protein Spidroin-2 [42]. Both Cnidoin and myxozoan Ncol-4 and Ncol-5 have glycine-rich regions next to the CRD, which is thought to be involved in network formation with other minicollagens [16]. Remarkably, the cysteine pattern (C…C..........C….C…..CC) of the C-terminal CRDs of Ncol-4 and Ncol-5 in the 9/10 residue spacing between C2 and C3 represents a common structural motif. As in cnidarian minicollagens, this might indicate the presence of intermolecular links between Ncol-4 and Ncol-5 generated by C-CRD cross-linking during myxospore development [22].

## Conclusions

In this study, we identified a novel myxozoan minicollagen (Ncol-5) by bioinformatic analysis of newly generated transcriptomes of two myxozoan species and published myxozoan NGS data. We assume, based on specific aspects of the gene structure, that Ncol-5 may play a specific role in the function of the polar capsule or tubule development. Our reconstruction of the minicollagen evolutionary history also suggests recent gene multiplications in Cnidaria, and indicates that myxozoan minicollagen repertoires have not been simplified by parasitic life strategy. The presence of Ncol-1 and Ncol-4 gene clusters in Myxozoa also indicates their common evolutionary history. Moreover, our novel bioinformatic pipeline improved by the filtering step of the coinfection by closely related species in transcriptome assembly will be a useful resource for such generations of transcriptomes in the future.

## Methods

### Myxozoan sampling and extraction of DNA and RNA

Ten individuals of the Northern pike *E. lucius* were obtained from the fish farm Rybářství Třeboň a.s. (Třeboň, Czech Republic) in November 2015. All fish were euthanized with an overdose of buffered MS-222. Urinary bladders were screened for the presence of plasmodia or myxospores of *M. lieberkuehni*, and kidney tissue was screened for the presence of xenoma structures caused by *N. pickii*, using an Olympus BX51 light microscope. *M*. *lieberkuehni* plasmodia and *N*. *pickii* xenoma stages were stored in 400 μL TNES urea buffer (10 mM Tris-HCl [pH 8], 125 mM NaCl, 10 mM EDTA, 0.5% SDS, and 4 M urea) for DNA analysis, or 100 μL RNAlater (Sigma-Aldrich, St. Louis, MO, USA) for RNA extraction. Samples collected for subsequent DNA extraction were stored at 4 °C, whereas RNA extraction samples were initially stored at − 20 °C and then moved to − 80 °C for long-term storage. Total genomic DNA was extracted using a standard phenol-chloroform extraction protocol. *M*. *lieberkuehni* plasmodia from the urinary bladder and *N*. *pickii* xenoma stages were homogenized and digested overnight with Proteinase K (50 μg mL^− 1^; Serva, Germany) at 55 °C. Extracted DNA was re-suspended in 50–100 μL^− 1^ DNase-free water and left to dissolve overnight at 4 °C. Collected samples stored in RNAlater were first thawed on ice, and then RNA was extracted using the Total RNA Isolation Kit (Macherey-Nagel, Germany). Concentration and purity of DNA and RNA samples were measured on the NanoDrop spectrophotometer.

### Transcriptome preparation and assembly

Prior to RNA sequencing, SSU rDNA was sequenced to confirm the species identity of *M. lieberkuehni* and *N*. *pickii*. PCR was performed using universal eukaryotic primers Erib1 and Erib10 for 18S rDNA [43] in the primary PCR, and specific primers for Myxozoa myxGP2F + ACT1R [44, 45] in the subsequent nested PCR (see Additional file 6: Table S1 for primer details). PCRs were carried out in a total volume of 25 μL consisting of 1× Taq Buffer, 250 μM of each dNTP, 10 pmol of each primer, 1 U Taq-Purple polymerase (Top-Bio, Czech Republic), 1 μL DNA (50–150 ng), and sterile distilled H_2_O. For universal eukaryotic primers, the following conditions were used: 95 °C/3 min; 30 cycles of 94 °C /50 s, 48 °C/50 s, and 72 °C/2 min; and 72 °C/10 min). For myxozoan-specific PCR primers, the following conditions were used: 95 °C/3 min; 33 cycles of 94 °C/40 s, 54 °C/50 s, and 72 °C/1 min 40 s; and 72 °C/10 min). PCR products were purified with the Gel/PCR DNA Fragments Extraction Kit (Geneaid Biotech Ltd., New Taipei City, Taiwan) and sequenced directly (SeqMe s.r.o., Czech Republic).

RNA of *M*. *lieberkuehni* plasmodia from urinary bladder and *N*. *pickii* from xenoma stages was sequenced commercially (BGI Genomics, China). Sequencing reactions were performed on an Illumina HiSeq™ 2000 instrument generating paired-end 100 bp reads.

Raw sequence data from the Illumina sequencing platform were checked for quality control using FASTQC (Babraham Bioinformatics). Quality and adapter trimming was done using Trimmomatic [46]. The presence of host contamination and contamination of transcriptomes by other parasites were evaluated by BLAST queries using a set of eukaryotic SSU rDNAs, including hosts and representatives of a different parasitic group, such as Myxozoa. We used the same pipeline for host–parasite filtration and assembly for both myxozoan species. For *N. pickii*, high-quality reads were first mapped to the host genome (*E. lucius*, Acc. No. GCA_011004845.1) using the SHRiMP-2.2.3 mapper with local alignment parameters [47, 48]. Unmapped reads were de novo assembled using the Trinity software package [49]. We performed the second filtration of parasite from host by BLASTing assembled transcripts against the host genome a second time. Transcripts with any host similarity were removed using a custom script. Assembly of the transcriptome of *M. lieberkuehni* was performed as described for *N. pickii*, using the host genome of *E. lucius* (AccNo: GCA_011004845.1) for filtration and a newly assembled *N. pickii* transcriptome, as the *M. lieberkuehni* transcriptome was found to be contaminated by *N. pickii*. The reads that did not map to the host genome or *N. pickii* transcriptome were assembled de novo using the same pipeline as for *M. lieberkuehni*, as described above. We performed post-assembly filtration against the host genome and *N. pickii* transcriptome using BLAST. All transcripts with host hits were removed, and all transcripts with more than 90% similarity to *N. pickii* were removed using a custom-made script (https://github.com/Seraff/bio_utils/blob/master/filter_contigs_by_hit_coverage.py) developed specifically to filter out closely related species.

### Transcriptomic mining and bioinformatic analyses of *Myxidium lieberkuehni* and *Nephrocystidium pickii* minicollagens

Assembled contigs of *M. lieberkuehni* and *N. pickii* were searched against prepared queries of minicollagen sequences. For identification of putative minicollagen homologs, we compiled queries including publicly available sequences of known myxozoan minicollagens, together with accessible cnidarian minicollagen sequences from the NCBI nr database (Additional file 1). Additional sequences were retrieved from the supplementary materials of [19]. To specify the mining procedure, we filtered out specific regions, including N and C-terminal regions of the CRD domains of each minicollagen group. Query sets including CRD domains were BLASTed against locally created platforms of myxozoan transcriptomic data using the Standalone BLAST software package with a different set of algorithms. For each search of the minicollagen protein group, an E-value threshold of 1e^− 5^ was adopted. A lower threshold was set to prevent possible misidentification due to the strict detection limit. The corresponding hits were then searched in the assembled *M. lieberkuehni* and *N. pickii* data using Command Prompt scripts, whereas low-scoring hits below the threshold were mined manually using Geneious v8.0.5 [50].

Transcripts containing putative minicollagen sequences were analyzed using the CD-Search-NCBI program [51] to validate the functional classification and domain architecture. The SignalP 4.1 web tool was used to determine the presence and location of the signal peptide and putative cleavage sites [52]. All identified contigs were subsequently translated into amino acids and queried against the NCBI nr database using BLASTp in a reciprocal BLAST search approach, using “Basic BLAST” categories in the NCBI BLAST web interface. Finally, matching sequences were retained, and identical contigs were pruned.

### Tracing of novel minicollagens in myxozoan and selected cnidarian NGS data

To date, all publicly available myxozoan and selected cnidarian genomic and transcriptomic data (Additional file 1) were analyzed to identify unknown minicollagen orthologs. For each Standalone BLAST orthology search, we used the same approach as in the *M. lieberkuehni* and *N. pickii* mining procedure. For each identified ortholog, minicollagen identity was verified by phylogenetic analysis, as described below.

### PCR amplification of *Myxidium lieberkuehni* and *Nephrocystidium pickii* minicollagens

Verification of minicollagen genes in *M*. *lieberkuehni* and *N*. *pickii* transcriptomes and identification of their introns were performed based on PCR of individual genes. Specific primers (Additional file 6: Table S2) were designed for *M*. *lieberkuehni* Ncol-1, 3, and 5 and *N*. *pickii* Ncol-5; because the sequence of *N*. *pickii* Ncol-3 is not complete, PCR was not performed for this gene. Individual specific primer regions were cross-checked with either Primer-BLAST-NCBI-NIH or the Design-Primer plugin in the Geneious v8.0.5 platform [50]. Best-fitting regions were checked for primer dimers and hairpin structures in Oligo analyzer 3.1|IDT (PrimerQuest, IDT, Coralville, IA, USA). Primers were synthesized commercially (Generi-Biotech s.r.o., Czech Republic).

We performed gradient PCRs at a wide range of annealing temperatures to amplify the targeted regions of individual minicollagens. For the PCR screening, AccuPower® HotStart PCR PreMix (BIONEER, South Korea) was used to prepare PCR reactions containing 10 μL HS-Taq premix, 0.5 μL of each primer (25 pmol), 8 μL nanopure H_2_O, and 1 μL DNA (50–150 ng). The following parameters were used for primary PCR of all identified minicollagen types: 94 °C/5 min; 35 cycles of 94 °C/30 s, 45–65 °C/1 min, 72 °C/1 min; and 72 °C/5 min. PCR products were purified using the Gel/PCR DNA Fragments Extraction Kit and commercially sequenced using SeqMe s.r.o.

### Alignment construction and phylogenetic analyses

Multiple sequence alignment of 128 minicollagen sequences (Additional file 1), omitting both N/C-terminal polyproline repeats, was constructed in MAFFT v7.450 [53] using the E-INS-i algorithm with the BLOSUM62 scoring matrix and default gap opening penalty. ML analysis was conducted through RAxML [54] using the best-scoring model (WAG) identified by ProtTest [55] and an LG model that refines the WAG model by incorporating the variability of evolutionary rates across sites into the matrix estimation [56]. RAxML trees were conducted using rapid hill climbing with the best-scoring tree topology search option. To obtain the best empirical expectations, the analysis started with a completely random tree. Bootstrap analysis was performed with 500 bootstrap replicates. BI analysis was conducted using MrBayes 3.2 [57] with the WAG model as the best-scoring model identified by ProtTest, with two runs with four chains each, sampling every 1000 generations, 25% burn-in, and termination when the standard deviation of split frequencies fell below 0.01.

### Ncol-5 CRD 3D structure comparison

3D protein structure comparison was performed to assess the 3D structural homology of newly identified myxozoan minicollagen with the published minicollagen of *H. vulgaris*. The minicollagen Ncol-5 N-terminal CRD protein sequences of *M. lieberkuehni* retrieved by PCR amplification were analyzed for the homology with published CRDs of Ncol-1 of *H. vulgaris* [58, 59]. To produce the 3D structure of selected CRD domains, two different software packages (Phyre 2, I-TASSER) were used [60, 61]. For the constructed tertiary structures, the published NMR structure of the Cys-rich N-terminal domain of *H. vulgaris* (1ZPX) deposited in the Protein Data Bank was used as a template. Stereochemical quality, compatibility of an atomic model of the 3D model with amino acid sequence (1D), and calculations of superpositions were evaluated in SuperPose, v1.0 [62], PROCHECK [63], and Verify 3D [64], respectively. 3D models were aligned and visualized in PyMOL (v2.0).

### Genomic localization of minicollagens

Genomic locations of minicollagen genes were evaluated using genomic data from Myxozoa, i.e., the genome of *T. kitauei* [65], *K. iwatai*, *S. zaharoni*, and *E. leei* [30]. Orthology blast hits of scaffolds with minicollagens were assembled de novo, and genes were predicted using Augustus implemented in Geneious v8.0.5 [50, 66]. Transcription direction was obtained from tab-delimited genome annotation files (*.gff). From data that lacked genome feature data, peptides encoded by transcripts were predicted by TransDecoder v5.5.0 (TransDecoder. https://transdecoder.github.io/), and transcriptional direction was assessed. Transcription direction of minicollagen peptides of species lacking corresponding RNA-seq data was determined from the genome using the Augustus prediction tool [66].

Close genomic juxtaposition of Ncol-1 and Ncol-4 of species with only transcriptomic data available (*S. molnari* and *C. shasta*) was demonstrated by PCR amplification. Newly designed sets of primers (Additional file 6: Table S3) targeting the genomic region encompassing Ncol-1 to Ncol-4 were used in gradient PCRs at a wide range of annealing temperatures with the following conditions: 94 °C/5 min; 35 cycles of 94 °C/30 s, 50–60 °C/1 min; and 72 °C/5 min. Purification and sequencing of the PCR products were performed as described above.

## Supplementary Information

**Additional file 1.**

**Additional file 2.**

**Additional file 3.**

**Additional file 4.**

**Additional file 5.**

**Additional file 6.**

## Data Availability

Datasets created or analyzed in this study have been deposited into NCBI under the BioProject accession PRJNA668274. All data generated from genomes and transcriptomes of Myxozoa and Cnidaria are summarized in Additional file 1, available from NCBI (https://www.ncbi.nlm.nih.gov/bioproject/).

## Abbreviations

TRGs: Taxonomically restricted genes
CRDs: Cysteine repeat domains
Cys-rich: Cysteine rich
N-CRD: N-terminal cysteine repeat domain
C-CRD: C-terminal cysteine repeat domain
Gly-X-Y: Glycine repeats
Poly-P: Polyproline
IGR: Noncoding intergenic region
MAFFT: Multiple alignment using fast fourier transform
BI: Bayesian inference
ML: Maximum likelihood
SRA: Sequence read archive
TSA: Transcriptome shotgun assembly
bp: Base pair
TNES: Tris, NaCl, EDTA, SDS
EDTA: Ethylenediaminetetraacetic acid
SDS: Sodium dodecyl sulfate
