# Supplementary material for: The myxozoan minicollagen gene repertoire was not simplified by the parasitic lifestyle: computational identification of a novel myxozoan minicollagen gene
Source: BMC Genomics. 2021 Mar 20;22:198. doi: 10.1186/s12864-021-07515-3 (PMC7981951; doi:10.1186/s12864-021-07515-3)
Supplement: Supplementary file 2 — Additional file 2. [file 12864_2021_7515_MOESM2_ESM.pdf]

Additional file 2

Cnidarian minicollagen poly-proline region at the N-terminal region

Sequence	Poly-proline region	Group
Hydra vulgaris Ncol-1	YPPPPPPPPPPPPPPPPPPPPPPPAPLP	Hydrozoa (*Hydra vulgaris*)
Hydra vulgaris Ncol-2	APPPPPPPPPPPPPPPPPPPPPPPVAIP	
Hydra vulgaris Ncol-3	MPPPPPPPPPPPPYP	
Hydra vulgaris Ncol-4	GPPPPPPPPPPPPPPIP	
Hydra vulgaris Ncol-5	YAQPAQPPPPPP	
Hydra vulgaris Ncol-6	IPQPPPPPP	
Hydra vulgaris Ncol-7	APPPPPPPPPALIIQ	
Hydra vulgaris Ncol-8	PPPPIYIPPPPPPPPIP	
Hydra vulgaris Ncol-9	SPPAPPPPAIPIP	
Hydra vulgaris Ncol-10	VPPPPPPPVSV	
Hydra vulgaris Ncol-11	VPPPPPPPPPPPPPPPPPPPPPPPPLPLP	
Hydra vulgaris Ncol-12	MAPPPPPPPPPLFLPPPPPPPPPPPPAPLP	
Hydra vulgaris Ncol-13	AMLPPPPPPPPPPCFCAPPPAPCGCGMPAPAPLP	
Hydra vulgaris Ncol-14	YPPPPPPPPPPPPPIP	
Hydra vulgaris Ncol-15	GGFAQMPCAPPPPPPQMIPMPVAMPAPCSPQMCGSQMPVGCSPQMCGQSYGQQMGGCGGGGYGGAQGGYGGSQPSYQVAYVQ	
Hydra vulgaris Ncol-16	PPPPPPPPPSPIVLPPTVIQIPFPPSYSPCGPSSPLPQMPCGASAPATAPCSSMSSNPAYSCIGANSMYPSGGASVIP	
Hydra vulgaris Ncol-17	AASMIMPPSPMCGSAGCSFGYPAAYPSSYSSMMMGMPSMVSPM	
Morbakka virulenta Ncol-1	APPPPPPPPPPPPPPPPPPPPPPPPPPPPPPPPPVP	Cubozoa (*Morbakka virulenta*)
Morbakka virulenta Ncol-2	YPPPPPPPPP	
Morbakka virulenta Ncol-3	VPAGPAPPPP	
Morbakka virulenta Ncol-4	AVAPPPPPPPPPPPPPPPPPPPPPPPPPPPQQPLP	
Morbakka virulenta Ncol-5	FPAAPPPPPP	
Morbakka virulenta Ncol-6	AAPPPAGPPPAAPPPPPPSPPKD	
Morbakka virulenta Ncol-7	SSGGYGGYGAAGAGCGGAAGGAGGAGCGGASYAGYGGGYGGGYGGGYAAPAPAPAPAPIP	
Nematostella vectensis Ncol-1	MAPPPPPPPPPVMCCAPPPPPPAMIP	Anthozoa (*Nematostella vectensis*)
Nematostella vectensis Ncol-3	LPPPPPPPPPPPPPPPPPEPARP	
Nematostella vectensis Ncol-4	SFTPPPPPPPPPYYPAPPPPP	
Nematostella vectensis Ncol-5	APPPPPPLPPLPPALPPPPPPPP	
Nematostella vectensis Ncol-6	APPPPPPPPPPPAQP	
Polypodium hydriforme Ncol-1	IPAPPPPPPPPP	*Polypodium* (*Polypodium hydriforme*)
Polypodium hydriforme Ncol-2	LPPPPPPPPP	
Polypodium hydriforme Ncol-3	IPAPPPPPPPPP	
Polypodium hydriforme Ncol-4	IPVPPPPPPPPP	
Polypodium hydriforme Ncol-5	VPPPPPPPVL	
Polypodium hydriforme Ncol-6	AQLQALPPPPPP	
Polypodium hydriforme Ncol-7	SGPGMIAA	
Polypodium hydriforme Ncol-8	GAGAGGYAAYPAPAPACGYASAPACAPAAAAAPMMIP	
Polypodium hydriforme Ncol-9	SYMINPVPPPPPPMAPTCMYPSSCYAPAPPACMASPMCASLPA	
Polypodium hydriforme Ncol-10	STPVQAPP	
Polypodium hydriforme Ncol-11	APAGPIPALP	
Sphaeromyxa zaharoni Ncol-1	YSMAAVPAVAAIPAVAAVPALPPLPPPPPP	Myxozoa (*Sphaeromyxa zaharoni*)
Sphaeromyxa zaharoni Ncol-2	AAPLPPLPPPPP	
Sphaeromyxa zaharoni Ncol-3	AAPAPAPVYVAPAPAPVYVPPPAPPVYVPPPPPPPPLPPLPPLPALP	
Sphaeromyxa zaharoni Ncol-4	GTGAMPIPPPPPPPTAVLIP	
Sphaeromyxa zaharoni Ncol-5	GIEGGSSSSSGSSSVSV	
