# Supplementary material for: The myxozoan minicollagen gene repertoire was not simplified by the parasitic lifestyle: computational identification of a novel myxozoan minicollagen gene
Source: BMC Genomics. 2021 Mar 20;22:198. doi: 10.1186/s12864-021-07515-3 (PMC7981951; doi:10.1186/s12864-021-07515-3)
Supplement: Supplementary file 3 — Additional file 3. [file 12864_2021_7515_MOESM3_ESM.docx]

**Additional file 3**

Minicollagen sequences of *Myxidium lieberkuehni*, *Nephrocystidium pickii*, *Sphaerospora molnari* and *Ceratonova shasta*. (A) Ncol-1; *Myxidium lieberkuehni*, (B) Ncol-3; *Myxidium lieberkuehni*, (C) Ncol-5; *Myxidium lieberkuehni*, (D) Ncol-3; *Nephrocystidium pickii*, (E) Ncol-5; *Nephrocystidium pickii,* (F) Ncol-1; *Sphaerospora molnari* (G) Ncol-1a; S*phaerospora molnari*, (H) Ncol-3; S*phaerospora molnari*, (I) Ncol-4; *Sphaerospora molnari*, (J) Ncol-5; *Sphaerospora molnari*, (K) Ncol-1; *Ceratonova shasta*, (L) Ncol-4; *Ceratonova shasta*, (M) Ncol-5;  *Ceratonova shasta* ). Gene architectures are highlighted (blue: signal peptide, yellow: intron positions, red: amino acid sequence).

A: *Myxidium lieberkuehni* Ncol-1

**ATGAAACATTTATCTTTAACTTTATTTGTGATTGGATTATCATGTGTCTTTGCA**GGCATGCCAAGATCAATTG**GTATTAATTTTAAATAATCTTAAATCCAATTTTTAAG**AAAAACGACAAACTTTTTGTTCAAATTCTTGCCCATCTTATTGTGCTCCAGCTTGCACACCAATGTGCTGTTATCCTCCAGCACTTCCACCACCTCCACCAGGTCCAATGGGTCAACCTGGYCCAACAGGACAAATGGGTCCTCCAGGACCCCCAGGACCTCCAGGACCACCAGGATTTCCAGGATCTATGGGATTAGCTGGATCACCAGGATATCCAGGCGCTGCTGCAGGACCTGCAGGACCAAACGGACAACCC**GTATATACATTTTTTTAATTATTCATTTCAAAG**GGACCAATGGGTCAACCAGGATTTCAAGGACAACCAGGGCCAAATGGACCTCAAGGACCCCCAGGACCACCAGGATTGCCAGGAGCTCCTGCACCACCACCACCTCCTCCACCATGTCCTTACGTTTGTACTACTACCTGTCTCCCATCTTGTCACCCAACATGCTGCAAGCAC**TAA**

MKHLSLTLFVIGLSCVFAGMPRSIEKRQTFCSNSCPSYCAPACTPMCCYPPALPPPPPGPMGQPGPTGQMGPPGPPGPPGPPGFPGSMGLAGSPGYPGAAAGPAGPNGQPGPMGQPGFQGQPGPNGPQGPPGPPGLPGAPAPPPPPPPCPYVCTTTCLPSCHPTCCKH

B: *Myxidium lieberkuehni* Ncol-3

**ATGTCATTATCCTCTTTGATTATCCTTTTCAGTTCCGTTGTTGTTTTAGCATTAACT**AAAAATATTGATG**GTATAATAATATCCTAATAATTTTTAAATTTCAATAG**GTGCTATTAAAAGAGATTTATATCCTTGTGGAGGATCATGCCCTTCATACTGTGCACCATCATGCAGTCAAATGTGYTGCTCTGCTCCTCCCCCAGTATATATTCCACCTCCACCTGCTCCACCTATTCCACCTGCGCCACCTCTTCCGGCATTACCTGGACCTCCAGGACCACCAGGAAGACCCGGACCTTCTGGATTAATGGGACCACCTGGAGCACCTGGACCACAAGGAGCTCCTGGAGCACCAGGTATGCCTGGAAGTCCTGGACAACCTGGAAGCCCAGCTCCACCACCAGCTCCATGTCCACCTATTTGCCAAACCCAATGTGTTATGGATTGCCCATTATACTGCTGTCCTTCTCGAAAATAA

MSLSSLIILFSSVVVLALTKNIDGAIKRDLYPCGGSCPSYCAPSCSQMCCSAPPPVYIPPPPAPPIPPAPPLPALPGPPGPPGRPGPSGLMGPPGAPGPQGAPGAPGMPGSPGQPGSPAPPPAPCPPICQTQCVMDCPLYCCPSRK

C: *Myxidium lieberkuehni* Ncol-5

**ATGGCTCGTATATTTTTGTCAACTTTTATTATATCAGTTATATTTATCATCGCAAGTATAAAT**TCCTTCCCAG**GTATATATTTTTTAACATTTTCAAGTTTTAAG**ATTATTGCGGAGGTGGATGTCCACTTATGTGTGCTCCAGGATGCTTACCAGGATGCTGCTTTGGCGGTTCTGGTGGAGGTTCTAACGGAGGTTCTAATGGAGGTTCCTCAGGAAGTAATGGAATGTCTGGACCACCCGGAATGCCTGGAAGTATGGGACTGATGGGTCCTCCAGGTCCTCCAGGACCTGCTGGACCTCAAGGACCTCCAGGATTACCTGGTCCTCAAGGACAACCTGGAATTCAGGGTAATGTTGGACCTGCTGGAAATCCTGGAATGACTGGACCTGCAGGAAGACCTGGACCTCAAGGACCTCCAGGAAGCAATAGCGCATCAGGCTCAAATGGTGGATATCCTGGTGAAAATAGTCAAGGTGGTTCTCAAGGATGTCCTAACGGTTGTGGACCAGGAATGTTCTTACTAACATCATGCCCATCCTTCTGTCCAAGTTATTGCTGTTAA

MARIFLSTFIISVIFIIASINSFPDYCGGGCPLMCAPGCLPGCCFGGSGGGSNGGSNGGSSGSNGMSGPPGMPGSMGLMGPPGPPGPAGPQGPPGLPGPQGQPGIQGNVGPAGNPGMTGPAGRPGPQGPPGSNSASGSNGGYPGENSQGGSQGCPNGCGPGMFLLTSCPSFCPSYCC

D: *Nephrocystidium pickii* Ncol-3 partial

ATAAAAAGAGATTTATATCCTTGTGGGATGTCATGCCCTCAATACTGTGCACCAGCTTGTACTCCAATGTGCTGTTCTGCACCTCAACCGATCTATGCACCACCACCACCACCTATTCCACCAGCCCCACCCCTACCAGCCTTACCCGGACCCCCAGGACCACCAGGAAGACCTGGACCATCTGGAATGATGGGACCACCTGGACAACCTGGACCACAAGGAGCTCCTGGTGCCCCTGGTATGCCTGGAAGCCCCGGACAACCTGGAAGCCCTGCTCCACCACCAGCTCCA

IKRDLYPCGMSCPQYCAPACTPMCCSAPQPIYAPPPPPIPPAPPLPALPGPPGPPGRPGPSGMMGPPGQPGPQGAPGAPGMPGSPGQPGSPAPPPAP

E: *Nephrocystidium pickii* Ncol-5

**ATGTTTCAGTCAGTTAGTATTCTATCAGCTTTATTTATCATTTCCACTATTTATTCTT**TGCCTG**GTAATATTTTAKTAGTGCTTTTGCAAGTATATGTATATAG**ATTTTTGTGGAGGTGGATGTCCTATGATGTGTGCGCCTGCATGTTTACCTGGATGCTGTCTAGGTGGATCTGGAGGATCAAATGGAGGAGGACCCGGTGGACAAGGAGGTAGCGGTCAATCAGGTCCACCAGGAATGCCTGGAAGTATGGGTTTAATGGGCCCTCCAGGACCACCAGGACCTCCAGGACCAGCTGGATCTCCGGGATTACCAGGACCTCAAGGACAGCCAGGAATACAAGGAAACGTTGGACCTGCGGGTAGTCCAGGAATGATGGGACCTGCAGGAAGACCAGGACCTCAAGGAAATCCGGGATATGCCGGTAGTCCAGGACCTTCAGGTGATTCAGGCCAAGGTGGTATGAGTGGTATGGGCGGTATGGGTGGAAGTGGTGGTATGAGCTGCCCTAACGGCTGTGGACCAGGAATGTTCCTCTTATCTGCATGCCCATCGTTCTGTCCAAGCTACTGCTGTTAA

MFQSVSILSALFIISTIYSLPDFCGGGCPMMCAPACLPGCCLGGSGGSNGGGPGGQGGSGQSGPPGMPGSMGLMGPPGPPGPPGPAGSPGLPGPQGQPGIQGNVGPAGSPGMMGPAGRPGPQGNPGYAGSPGPSGDSGQGGMSGMGGMGGSGGMSCPNGCGPGMFLLSACPS

F: *Sphaerospora molnari* Ncol-1

**ATGGCTTGCAACCTTGTTTTGTTGTTACTTCTGGTAGGACCAGCTATTGCT**AGTCTTCCTAGCAAAATTG**GTAAGAAAATAAACCAAATTTTTAAATATCCATGCACCAG**ATAAAAGAAGTCCCCAAATGACTTGTGGGTCTCCTTGTCCAGCTTACTGTGCTCCAGCTTGCAGTCCAGTATGTTGCGTGTCTCCACTACCCCCACCACCTCCAGGACCAGCTGGAGCTCCAGGAGCTGCAGGTGCTCCAGGAGCCCAAGGACCACCTGGAATTCCTGGTCCAAACGGACCACCTGGAGTTCCTGGTGCTGCTGGTGCTCCCGGATTTCCTGGAGCAGCTGCAGGGCCACAAGGACCTAACGGACCACAA**GTACTAGTTTTATAAATTTAAATTAAACTTCTTCTCAATTCAG**GGACCTAATGGAGCCATGGGACAGCCCGGACCAATGGGTCTTCCCGGTATGCCTGGACCACCAGGTCCACCAGGAGCTCCCGGTGCACCAGCTCCACCTCCACCACCTCCCCCATGTCCTTACATTTGCACTCAAACATGTACCAAGACTTGTCACCCAACGTGCTGTGCCAAGAAC

MACNLVLLLLLVGPAIASLPSKIDKRSPQMTCGSPCPAYCAPACSPVCCVSPLPPPPPGPAGAPGAAGAPGAQGPPGIPGPNGPPGVPGAAGAPGFPGAAAGPQGPNGPQGPNGAMGQPGPMGLPGMPGPPGPPGAPGAPAPPPPPPPCPYICTQTCTKTCHPTCCAKN

G: *Sphaerospora molnari* Ncol-1a

**ATGAGCATCCTGTTGACGTTGGCTACGTTACTTTTTACAGTCCAGGCT**ACTTTGCCTACCACTAAAGAGAAACGCCAAGCAACTTGCGGAGTTGCCTGTCCGAGCTATTGCGCACCGGCTTGTACTCCAGTTTGTTGCTCTCCTCTTCCACCTCCACCTCCAGGACCTGTTGGCGCAGCTGGACAAGCTGGACCTCAAGGACCATCTGGACCACCAGGCCCACCCGGACCACCAGGACCACCTGGTGTTTCAGGCGGAGCTGGAGCTCCCGGATATCCTGGTGCCGCTGCAGGACCTCAAGGACCAAACGGACCAGCTGGGCCAATTGGACAAATGGGACAACCCGGACCTCAAGGCCTTCCTGGTAAACCAGGTATGCCAGGACCACCAGGAGCACCTGGAGCCCCAGCACCACCTCCCCCACCTCCTCCCTGTCCTTACATCTGTACCCTACAGTGTACCAATACTTGCCATCCAACCTGCTGCAAGCAC**TAG**

MSILLTLATLLFTVQATLPTTKEKRQATCGVACPSYCAPACTPVCCSPLPPPPPGPVGAAGQAGPQGPSGPPGPPGPPGPPGVSGGAGAPGYPGAAAGPQGPNGPAGPIGQMGQPGPQGLPGKPGMPGPPGAPGAPAPPPPPPPCPYICTLQCTNTCHPTCCKH

H: *Sphaerospora molnari* Ncol-3

**ATGAGGTCCGAAGTTGCAATCTGTCTTTTGCTGGGACTTGTGTCCGTGTACAGCTCC**AACGTTATTGATAAGCTTTTCAAACGTTCCCCACAATATGGAGCATGTGGAGCTACATGTCCATCATATTGTGCACCTGCTTGTACTCCTCAGTGTTGTACTGTAGCACCCCCACCACCACCACCAGTGTACATTCCACCTCCTCCACCTCCACCACCACCCCCACTTCCAGCTCTTCCTGGACCACCAGGACCACCCGGTAGACCAGGACCATCCGGAGCACCAGGACCTCAAGGACCAGCTGGACCACCAGGCCCACCTGGAGCACCAGGTTTGGCAGGAACTCCAGGCATGCCTGGAGCTCCAGCTCCACCACCAGCCCCTTGCCCACCAATTTGTGCTCAACAGTGTGTCACTCAGTGTCCTCTGTATTGCTGTCCCGCCAAGAGAAGA

MRSEVAICLLLGLVSVYSSNVIDKLFKRSPQYGACGATCPSYCAPACTPQCCTVAPPPPPPVYIPPPPPPPPPPLPALPGPPGPPGRPGPSGAPGPQGPAGPPGPPGAPGLAGTPGMPGAPAPPPAPCPPICAQQCVTQCPLYCCPAKRR

I: *Sphaerospora molnari* Ncol-4

**ATGGGTCCTTATTTTAAATCAATAAG**CATTCTCTCAGAAG**GTAACCACAATCGACCATCAGTCCAGCTTGAACAAAAACACTATTTGTGGATGATGATGATGTTTTTCATTCTACAATTTTTCCTATTATCCGCACATTCGCTGCAAGTTGGTAAATGAAGTATTACTTCAAATATTTTATTTAATTATCCCCAG**AGAATGTCAGAAAAAGATCCGCTCAAGTTTGTGGATATGGTTGTCCATCGTCTTGTTATCCAGCATGTACGGCATCATGTTGTTCCAATACACCTACATACCAACCTCCTCCACCTGTACCTGCTCCAGTCGTAATTCAGACTATTGCAGCACCGCCCACTTCCTCGGCTCTTCCAGTTGCTCCACCGATTTCATATTCAGTATCTGTTCCAATTGCCCCACCACCACCTCCCCCTCAGCCTCCAGCAATAATGCAACCTCAGTCAATCGCTCAGTGTAGCTCAGCTAATTGTGGACCACCTCCGGTTCCACCACCAACTGTGATATTTAGTGGACCTCCTGGGCCGCCTGGTCCACCAGGTCTTCCAGGTGGACAAGGTCCAACACCGCCTGCGGGATGCCCTCCAATTTGCATTACAACCTGCATTAAACCTTGTCCACTTCAATGTTGTGCTTCTCCATCTGTCGCAACGCCTCCAACACCACCTCCAGTTACAGGATCATATGCATGTCCTCCCTCCCAAGTATGTGGACCACCAGCCCCACAAGTTATTGCAGCCCCTCCACCTCCAGTAATTTATCAGGTTCCACAATATGCTCCTCCTGCACCTGCTCCAACATCCATTATTTGCCGACCCTGTGTGGCACCCCAAAAACCCCAAGTGATGTGTTCATATTATGGATGTGCACCGACATTGTGTTGC**TGA**

MGPYFKSISILSEENVRKRSAQVCGYGCPSSCYPACTASCCSNTPTYQPPPPVPAPVVIQTIAAPPTSSALPVAPPISYSVSVPIAPPPPPPQPPAIMQPQSIAQCSSANCGPPPVPPPTVIFSGPPGPPGPPGLPGGQGPTPPAGCPPICITTCIKPCPLQCCASPSVATPPTPPPVTGSYACPPSQVCGPPAPQVIAAPPPPVIYQVPQYAPPAPAPTSIICRPCVAPQKPQVMCSYYGCAPTLCC

J: *Sphaerospora molnari* Ncol-5

**ATGAATTTAAATGTGTTGGTTTATGGAATTTTTTCCATGATATTAATGAGCTGTCCTTGGAATATAGTAGCA**TTTGAAACTTGTGGTTCTGGTTGTCCTTTGATTTGTGCTCCGGCATGTCTCCCAGGATGTTGCTTCGGAGGTAGTGGTGGTGGTGGTGGTGGTTCACCTTCTTCATCAGGTTACGGTTCTGCCTCTAGTTCAAGTGGTGCCTCATCTTCATCAAGTGGGGCCCAAGGAGCGCAAGGAATGTCTGGACCACCAGGAATGCCTGGAGCTATGGGTTTAATGGGACCATCTGGTCCACCTGGACCACCAGGCCCACCCGGACCTCCTGGACAACCTGGTACTCAAGGACCACCTGGTATTGCGGGACCAATGGGTCCTGTTGGACCAGTTGGACCATCTGGACCGGTTGGTCGACCAGGACAACCTGGAGCTGCTGGTATGCCTGGGTCTGCTGGCAGTTACGGAGATTCATCAAGTGCTGGCGCTTCAAGCGCCGCAGCTTCATCAGCTTCTGGAGCTTCAGCTGGCAGAGGTGGTCTAGGATGTCCTGCAGGATGTACTCCTGGAATGTATTTGATTACGGCCTGTCCTTCTTTCTGTCCATCATATTGCTGT

MNLNVLVYGIFSMILMSCPWNIVAFETCGSGCPLICAPACLPGCCFGGSGGGGGGSPSSSGYGSASSSSGASSSSSGAQGAQGMSGPPGMPGAMGLMGPSGPPGPPGPPGPPGQPGTQGPPGIAGPMGPVGPVGPSGPVGRPGQPGAAGMPGSAGSYGDSSSAGASSAAASSASGASAGRGGLGCPAGCTPGMYLITACPSFCPSYCC

K: *Ceratonova shasta* Ncol-1

**ATGGTTGTTGTAGTCGTACCAATA**ATATTTGTACTATTCTCTTATGTTTCTTCCGGATTACCAAAATCAATGA**GTAAGATCATTTGTTGATAAATAAAG**ATAAAAGAAGTCCAGGAATGTGTGCACCAGCTTGTCCAGCTTTATGTGCTCCAGTTTGTTCTGATAATTGTTGTTATGCATCACCATTGCCACCCCCACCACCAGGTCCACCAGGAATAATGGGAGCACCAGGATTAGCTGGTCCTAATGGACCCTCAGGACCACCTGGACCTCCTGGACCCCCAGGTATTGGAGGTTTTCCAGGATCACCAGGATATCCAGGTGCAATGGCCGGACCACCAGGAATAAACGGATTACCA**GTATTAAAATTTAAAAATAATAG**GGACCAATGGGAGGAAGAGGAAATTCCGGACCACAAGGACCTCAAGGATTACAAGGACCACCAGGTCCACCAGGAGCTCCAGGTGCACCAGCTCCACCACCCCCACCAACTCCATGTCCAGCTTTATGTTTGACAACTTGTACAACTAGTTGTCATCCAACATGCTGTAAACATTAA

MVVVVVPIIFVLFSYVSSGLPKSMNKRSPGMCAPACPALCAPVCSDNCCYASPLPPPPPGPPGIMGAPGLAGPNGPSGPPGPPGPPGIGGFPGSPGYPGAMAGPPGINGLPGPMGGRGNSGPQGPQGLQGPPGPPGAPGAPAPPPPPTPCPALCLTTCTTSCHPTCCKH

L: *Ceratonova shasta* Ncol-4

**ATGAGAAGTTTGATATTATTTTTATTTTTGGAGCAAATAATAAGT**TATAGTTTAA**GTAAATTTATCTAAGTTTGTTAG**ACAAAAGAAGTCCTCAACTATGTCCTTATCCGTGTAATTCTATTTGTGCCCCTCAGTGTAATCCACAATGTTGTAATGGAGGAGGTAATTCAGGAGGAGGAGGATTACCAAATACAGCTGTCTTGGTATCAGGTCCTCCAGGACCTCCTGGACCTCCTGGGATGTTAATGAAACAACCATGCAACCCTATATGTATTACAACCTGTATTAAAGAGTGTCCACCTCAGTGTTGTCAGCCAGGAGGAGGTTTACCAATGTGTTCACCTCAACAATGTGCTATGCCCAATTCGCCATGCTTTCCTCAACAATGTCAACCACAAATGCCACCTATGTGTTCTCCATCTCAATGCATGATGCCCAACTCACCTTGTATGCCACAACAATGTCCTCCACCTTTATCGAATATGTGTTCACCATCTCAGTGTATGATGCCCAACACACCTTGTTTGCCACAACAATGCCCTCCACCATCTGGACAATGTTCACCTGCTCAATGTATGATGCCCAACACTCCATGTATGCCACAACAATGTTTACAACTTTCAGGAATGTGTTCACCAGCTCAATGTACTGTACCTAATAATCAATGCAATCCGCAGCAGTGTCAAAATTTGCTCAAACAGATGATTTGCCCCAATCCTCCATGTTATCCCCAATCTAATCCTACTTGTATACCTTGCATGCCAAACCAACCTCCTCCAAAACAGCAATGTTTTCCAAATGGATGTATGCCTGCTCCAATTTGTTGCTAA

MRSLILFLFLEQIISYSLNKRSPQLCPYPCNSICAPQCNPQCCNGGGNSGGGGLPNTAVLVSGPPGPPGPPGMLMKQPCNPICITTCIKECPPQCCQPGGGLPMCSPQQCAMPNSPCFPQQCQPQMPPMCSPSQCMMPNSPCMPQQCPPPLSNMCSPSQCMMPNTPCLPQQCPPPSGQCSPAQCMMPNTPCMPQQCLQLSGMCSPAQCTVPNNQCNPQQCQNLLKQMICPNPPCYPQSNPTCIPCMPNQPPPKQQCFPNGCMPAPICC

M: *Ceratonova shasta* Ncol-5

**ATGTTGGGGTTGTTATGTTGTATTAGTTTGTTCACTTTATTATTAAATTATTTGAGTGTTGTAAATTCT**TTTCCAAATTTTTGCGGTCAAGGTTGTCCATCAATATGTGCACCTCAATGTAATGCTATGTGTTGCGACATTAGTCCAAAAACTTCTAGTTCTGGGTCTGCAGGACCACCAGGAATGCCAGGATCAATGGGATTAATGGGACCTCCTGGACCTCCAGGGCCTCCTGGTAATCCAGGTACACCAGGATCACCAGGAATGCAAGGACCCATGGGTCCTGTAGGGCCATCAGGACCAATGGGACCACCAGGTTCAGCAGGACTAATGGGTCCTCCAGGATTATCAGGAACTCCTGGAGTAGGAGGTTCGTCAGGAAACCCAGGGATAAATGGAAATCCAGGATCTCAAGTAGGTTCACAAGAGTCTTGTCCAAGTGGATGTACACCTGGGTTATATTTATTATCTCAATGCCCATCTATGTGCCCATCGCGTTGCTGTAG

MLGLLCCISLFTLLLNYLSVVNSFPNFCGQGCPSICAPQCNAMCCDISPKTSSSGSAGPPGMPGSMGLMGPPGPPGPPGNPGTPGSPGMQGPMGPVGPSGPMGPPGSAGLMGPPGLSGTPGVGGSSGNPGINGNPGSQVGSQESCPSGCTPGLYLLSQCPSMCPSRCC

Sequences of gene cluster of Ncol-1 and Ncol-4 of *Sphaerospora molnari* (A) and *Ceratonova shasta* (B). Gene architectures are highlighted (light blue: signal peptide, yellow: intron positions, grey: non coding sequence).

A: *Sphaerospora molnari* Ncol-1 + Ncol-4

TCAGCAACACAATGTCGGTGCACATCCATAATATGAACACATCACTTGGGGTTTTTGGGGTGCCACACAGGGTCGGCAAATAATGGATGTTGGAGCAGGTGCAGGAGGAGCATATTGTGGAACCTGATAAATTACTGGAGGTGGAGGGGCTGCAATAACTTGTGGGGCTGGTGGTCCACATACTTGGGAGGGAGGACATGCATATGATCCTGTAACTGGAGGTGGTGTTGGAGGCGTTGCGACAGATGGAGAAGCACAACATTGAAGTGGACAAGGTTTAATGCAGGTTGTAATGCAAATTGGAGGGCATCCCGCAGGCGGTGTTGGACCTTGTCCACCTGGAAGACCTGGTGGACCAGGCGGCCCAGGAGGTCCACTAAATATCACAGTTGGTGGTGGAACCGGAGGTGGTCCACAATTAGCTGAGCTACACTGAGCGATTGACTGAGGTTGCATTATTGCTGGAGGCTGAGGGGGAGGTGGTGGTGGGGCAATTGGAACAGATACTGAATATGAAATCGGTGGAGCAACTGGAAGAGCCGAGGAAGTGGGCGGTGCTGCAATAGTCTGAATTACGACTGGAGCAGGTACAGGTGGAGGAGGTTGGTATGTAGGTGTATTGGAACAACATGATGCCGTACATGCTGGATAACAAGACGATGGACAACCATATCCACAAACTTGAGCGGATCTTTTTCTGACATTCT**CTGGGGATAATTAAATAAAATATTTGAAGTAATACTTCATTTACCAACTTGCAGCGAATGTGCGGATAATAGGAAAAATTGTAGAATGAAAAACATCATCATCATCCACAAATAGTGTTTTTGTTCAAGCTGGACTGATGGTCGATTGTGGTTAC**CTTCTGAGAGAATGCT**TATTGATTTAAAATAAGGACCCAT**TTAATTATCTGCATTTATAACAATAAACACAAAATGTGATATTGGCAGGATTTGAATTGAATGGAATCTAATTATATGCATCACTTGGTTTATTTCAATCAGAAAAACCTGTGGAACGTCAATAAATTTGTTGTAACATTCAATTCGATTGAGTGCGTAGATTGCTAG**ATGGCTTGCAACCTTGTTTTGTTGTTACTTCTGGTAGGACCAGCTATTGCT**AGTCTTCCTAGCAAAATTG**GTAAGAAAATAAACCAAATTTTTAAATATCCATGCACCAG**ATAAAAGAAGTCCCCAAATGACTTGTGGGTCTCCTTGTCCAGCTTACTGTGCTCCAGCTTGCAGTCCAGTATGTTGCGTGTCTCCACTACCCCCACCACCTCCAGGACCAGCTGGAGCTCCAGGAGCTGCAGGTGCTCCAGGAGCCCAAGGACCACCTGGAATTCCTGGTCCAAACGGACCACCTGGAGTTCCTGGTGCTGCTGGTGCTCCCGGATTTCCTGGAGCAGCTGCAGGGCCACAAGGACCTAACGGACCACAA**GTACTAGTTTTATAAATTTAAATTAAACTTCTTCTCAATTCAG**GGACCTAATGGAGCCATGGGACAGCCCGGACCAATGGGTCTTCCCGGTATGCCTGGACCACCAGGTCCACCAGGAGCTCCCGGTGCACCAGCTCCACCTCCACCACCTCCCCCATGTCCTTACATTTGCACTCAAACAT

B: *Ceratonova shasta* Ncol-1 + Ncol-4

AACTAGTTGTACAAGTTGTCAAACATAAAGCTGGACATGGAGTTGGTGGGGGTGGTGGAGCTGGTGCACCTGGAGCTCCTGGTGGACCTGGTGGTCCTTGTAATCCTTGAGGTCCTTGTGGTCCGGAATTTCCTCTTCCTCCCATTGGTCC**CTATTATTTTTAAATTTTAATAC**TGGTAATCCGTTTATTCCTGGTGGTCCGGCCATTGCACCTGGATATCCTGGTGATCCTGGAAAACCTCCAATACCTGGGGGTCCAGGAGGTCCAGGTGGTCCTGAGGGTCCATTAGGACCAGCTAATCCTGGTGCTCCCATTATTCCTGGTGGACCTGGTGGTGGGGGTGGCAATGGTGATGCATAACAACAATTATCAGAACAAACTGGAGCACATAAAGCTGGACAAGCTGGTGCACACATTCCTGGACTTCTTTTAT**CTTTATTTATCAACAAATGATCTTAC**TCATTGATTTTGGTAATCCGGAAGAAACATAAGAGAATAGTACAAATAT**TATTGGTACGACTACAACAACCATTTTAAACT**TTGTATATAGAAGCTATTTTTTAGAACTTTAATAAAATTAAAATTATAATAGAGGCTTGTTTTCATTCAAAACACAAATTTATTTTTTAAATTTTAAGCTTGAACTTAAAGCATCTTTTCAATTGTAGCTAGATATTATATTCTCTGGATATTTCAAGCTTAATAAAAGTTTCATTAAGTAAGTGTTTTAGATTTAATATTGAAATTAAGA**ATGAGAAGTTTGATATTATTTTTATTTTTGGAGCAAATAATAAGT**TATAGTTTAA**GTAAATTTATCTAAGTTTGTTAG**ACAAAAGAAGTCCTCAACTATGTCCTTATCCGTGTAATTCTATTTGTGCCCCTCAGTGTAATCCACAATGTTGTAATGGAGGAGGTAATTCAGGAGGAGGAGGATTACCAAATACAGCTGTCTTGGTATCAGGTCCTCCAGGACCTCCTGGACCTCCTGGGATGTTAATGAAACAACCATGCAACCCTATATGTATTACAACCTGTATTAAAGAGTGTCCACCTCAGTGTTGTCAGCCAGGAGGAGGTTTACCAATGTGTTCACCTCAACAATGTGCTATGCCCAATTCGCCATGCTTTCCTCAACAATGTCAACCACAAATGCCACCTATGTGTTCTCCATCTCAATGCATGATGCCCAACTCACCTTGTATGCCACAACAATGTCCTCCACCTTTATCGAATATGTGTTCACCATCTCAGTGTATGATGCCCAACACACCTTGTTTGCCACAACAATGCCCTCCACCATCTGGACAATGTTCACCTGCTCAATGTATGATGCCCAACACTCCATGTATGCCACAACAATGTTTACAACTTTCAGGAATGTGTTCACCAGCTCAATGTACTGTACCTAATAATCAATGCAATCCGCAGCAGTGTCAAAATTTGCTCAAACAGATGATTTGCCCCAATCCTCCATGTTATCCCCAATCTAATCCTACTTGTATACCTTGCATGCCAAACCAACCTCCTCCAAAACAGCAATGTTTTCCAAATGGATGTAT
