# Supplementary material for: The myxozoan minicollagen gene repertoire was not simplified by the parasitic lifestyle: computational identification of a novel myxozoan minicollagen gene
Source: BMC Genomics. 2021 Mar 20;22:198. doi: 10.1186/s12864-021-07515-3 (PMC7981951; doi:10.1186/s12864-021-07515-3)

Additional file 4

Cys-rich protein domain homology and superposition of Ncol-5 of *Myxidium lieberkuehni* and Ncol-1 *Hydra vulgaris*

(A)

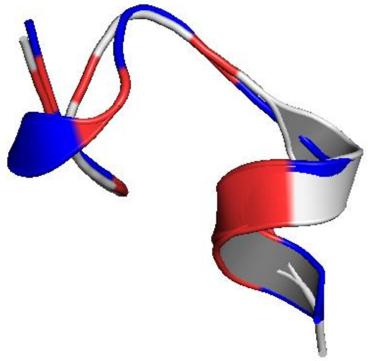

N-CRD

```
1 PCGSYCPSVCAPACAPVCCYP---------     21
  .||..||.:|||.|.|.||:.
1 YCGGGCPLMCAPGCLPGCCFGGSGGGSNGG     30
```

(B)

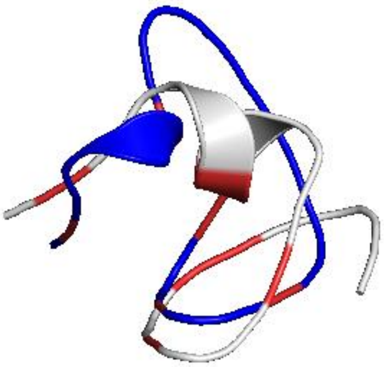

(C-CRD)

```
1 PCPPVC------VAQCVPTCPQYCCPAKRK     24
  .||..|      :..|...||.|||
1 GCPNGCGPGMFLLTSCPSFCPSYCC-----     25
```

Backbone ribbon representations of N-terminal (A) and C-terminal (B) Cys-rich domains of Ncol-5 of *Myxidium lieberkuehni* (blue) with superposition of N-CRD (1ZPX) and C-CRD (1SOP) of Ncol-1 of *Hydra vulgaris* (grey). Cysteine residues are highlighted in red. Alignments between the N-CRD and C-CRD of Ncol-1 protein structures from *H. vulgaris* and *M. lieberkuehni* generated by SuperPose are given under the backbone ribbon structure.

Ramachandran plot provided by the PROCHECK program for the 3D model of N-CRD (A) and C-CRD (B) protein structures of Ncol-5 of *M. lieberkuehni*.

(A)

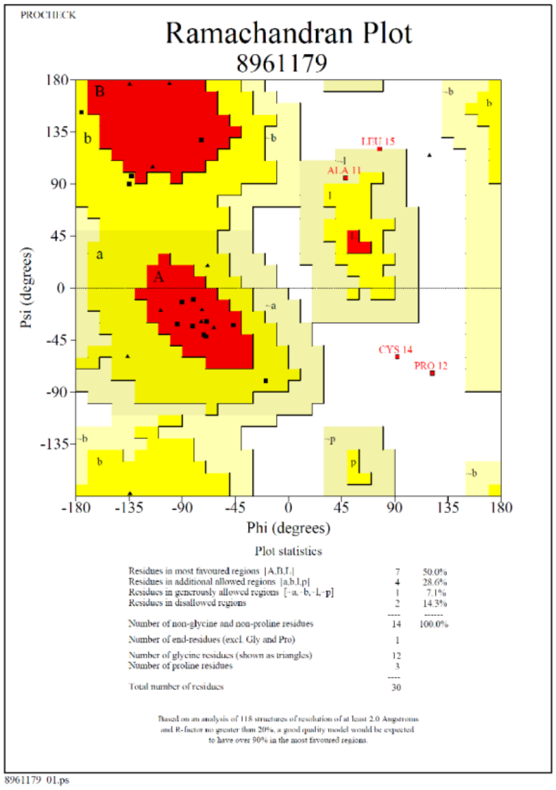

(B)

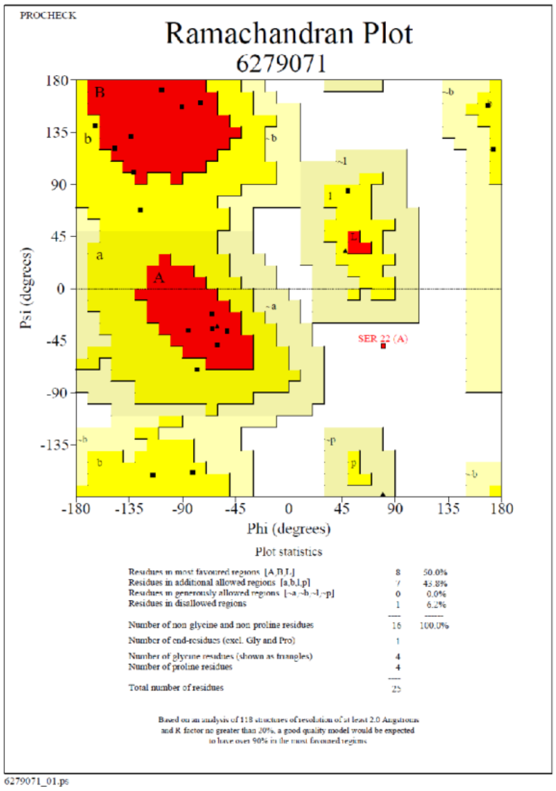

Compatibility of an atomic model (3D) with its own amino acid sequence (1D) of N-CRD (A) and C-CRD (B) protein structures of Ncol-5 of *M. lieberkuehni* generated in Verify3D.

(A)

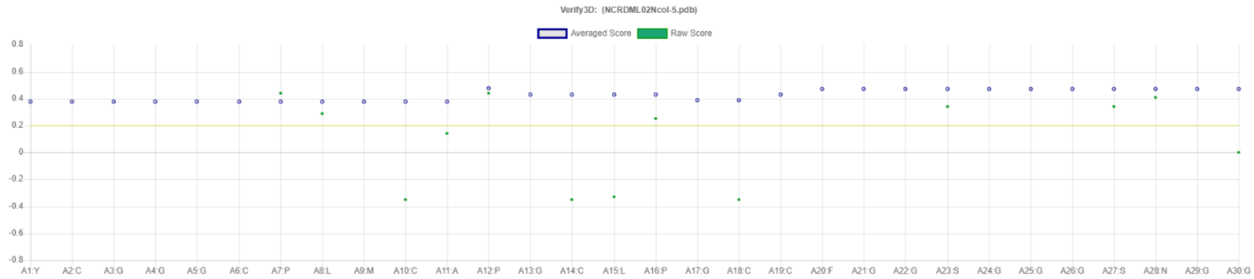

(B)

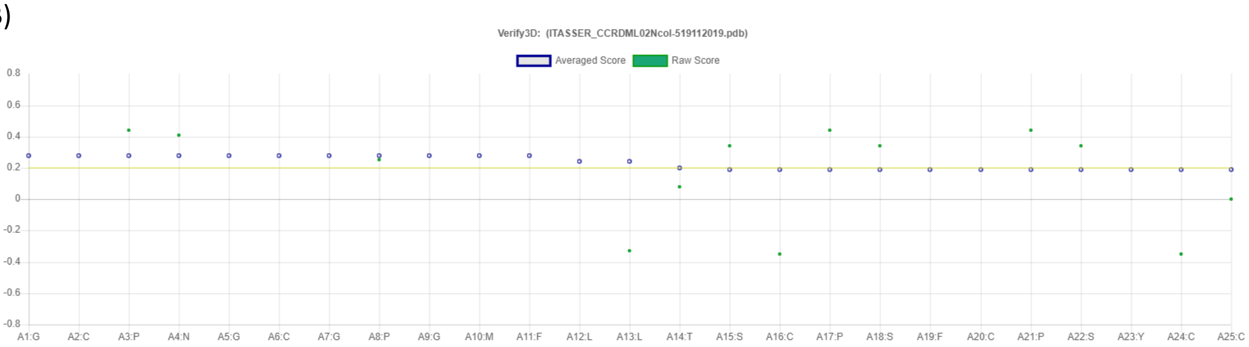

Supplement: Supplementary file 4 — Additional file 4. [file 12864_2021_7515_MOESM4_ESM.pdf]
