# Supplementary material for: The myxozoan minicollagen gene repertoire was not simplified by the parasitic lifestyle: computational identification of a novel myxozoan minicollagen gene
Source: BMC Genomics. 2021 Mar 20;22:198. doi: 10.1186/s12864-021-07515-3 (PMC7981951; doi:10.1186/s12864-021-07515-3)

Additional file 5

Maximum likelihood analysis of cnidarian minicollagen genes; LG model

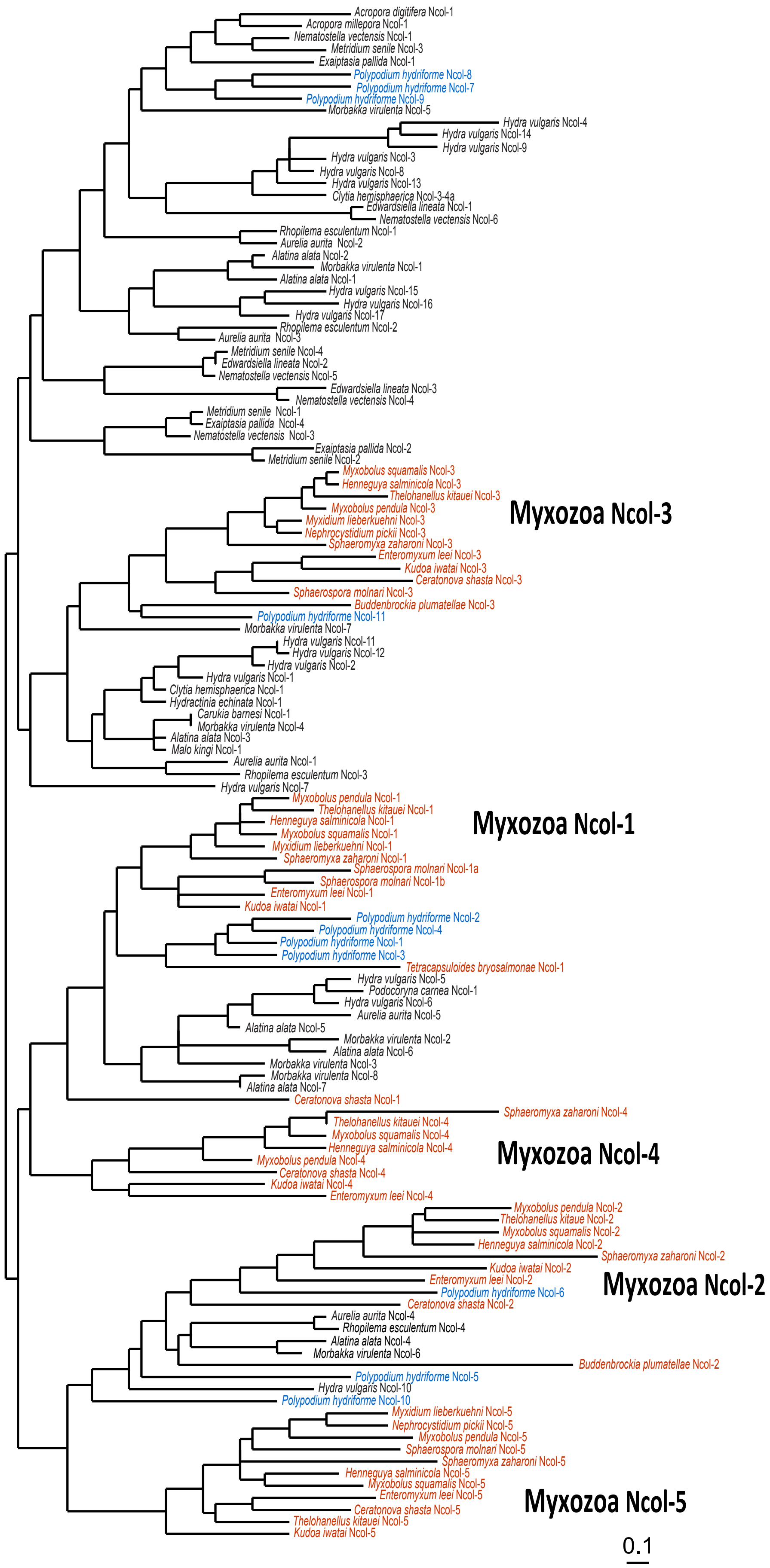

Maximum likelihood analysis of cnidarian minicollagen genes; WAG model

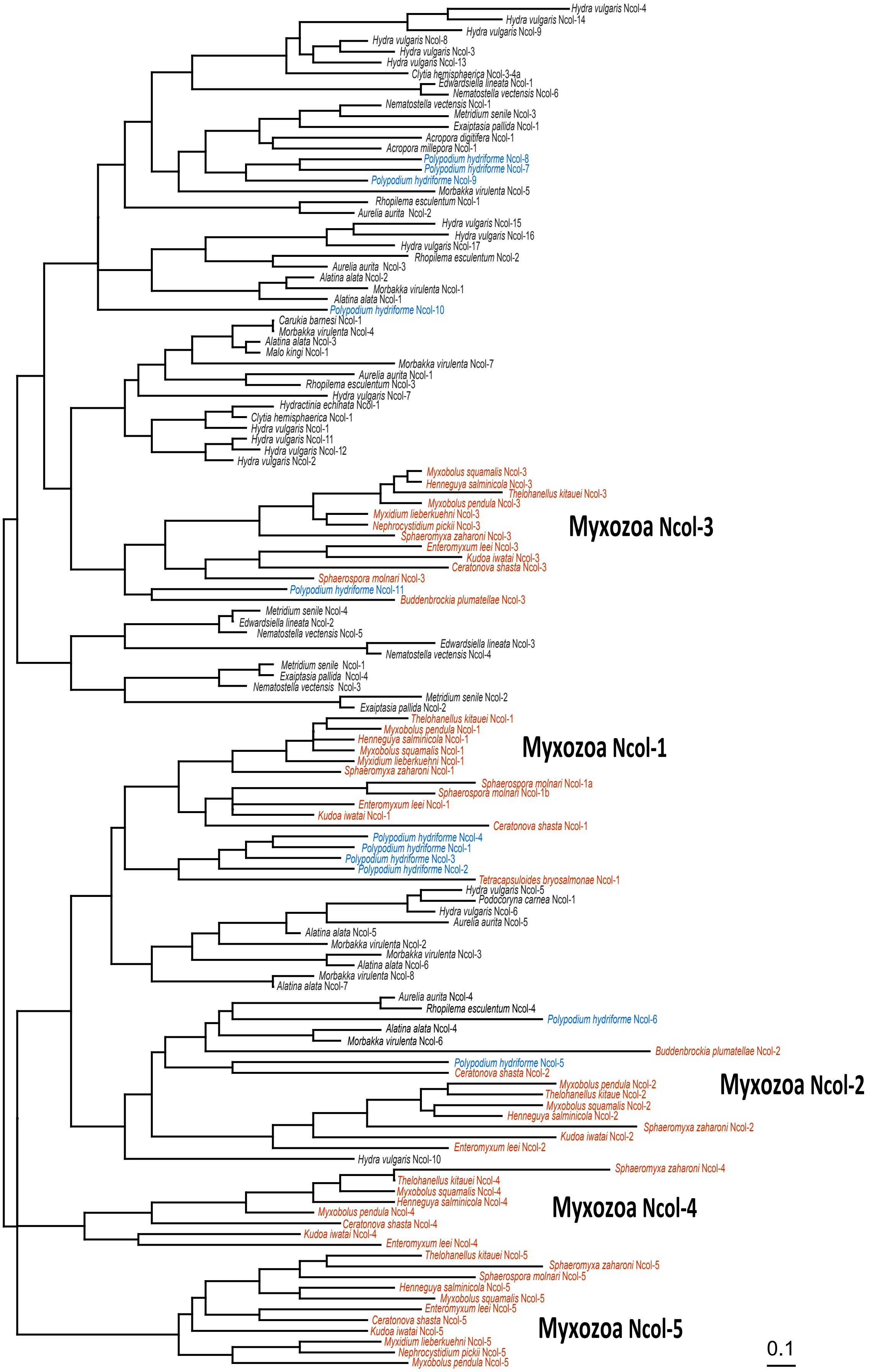

Supplement: Supplementary file 5 — Additional file 5. [file 12864_2021_7515_MOESM5_ESM.pdf]
