# Supplementary material for: The myxozoan minicollagen gene repertoire was not simplified by the parasitic lifestyle: computational identification of a novel myxozoan minicollagen gene
Source: BMC Genomics. 2021 Mar 20;22:198. doi: 10.1186/s12864-021-07515-3 (PMC7981951; doi:10.1186/s12864-021-07515-3)
Supplement: Supplementary file 6 — Additional file 6. [file 12864_2021_7515_MOESM6_ESM.docx]

**Additional file 6: Table S1**

List of primers used for SSU rDNA amplification

| **Primer name** | **Sequence** (5’→3’) | **PCR type** | **Tm (°C)** | **Reference** |
| --- | --- | --- | --- | --- |
| Erib1 | ACCTGGTTGATCCTGCCA | 1. run | 48 | Barta et al. (1997) |
| Erib10 | CTTCCGCAGGTTCACCTACGG |  |  |  |
| MyxGP2F | WTGGATAACCGTGGGAAA | 2. run | 54 | Kent et al. (1998) |
| ACT1R | AATTTCACCTCTCGCTGCCA |  |  | Hallet and Diamant (2001) |

**Additional file 6: Table S2**

List of primers used for minicollagen sequence verification and intron identification in *M. lieberkuehni* and *N. pickii*

| **Primer name** | **Sequence** (5’→3’) | **Specific for** | **Tm (°C)** | **Reference** |
| --- | --- | --- | --- | --- |
| ML_Ncol-1-F | AGATTGGACAAGATAAAATG | *Myxidium lieberkuehni*, Ncol-1 | 45 - 65 | This study |
| ML_Ncol-1-R | GATAAGTCCTTTGAATTTTA |  |  |  |
| ML_Ncol-3-F | GCGGAGAGAAACTATGTCAT | *Myxidium lieberkuehni*, Ncol-3 | 45 - 65 | This study |
| ML_Ncol-3-R | GTTAAGCGTTATTGATTTTTT |  |  |  |
| ML_Ncol-5-F | GTGATAAAATTCATTTACGG | *Myxidium lieberkuehni*, Ncol-5 | 45 - 65 | This study |
| ML_Ncol-5-R | TATAAGGAGATTTGAAATTTC |  |  |  |
| NP_Ncol-5-F | GCATCACACAAATGACTCAT | *Nephrocystidium pickii*, Ncol-5 | 45 - 65 | This study |
| NP_Ncol-5-R | TTAATCTGACATAGTTTA |  |  |  |

**Additional file 6: Table S3**

List of primers used for genomic localization of Ncol-1 and Ncol-4

| **Primer name** | **Sequence** (5’→3’) | **Specific for** | **Tm (°C)** | **Reference** |
| --- | --- | --- | --- | --- |
| CS_Ncol1-4-F | TAATGTTTACAGCATGTTGG | *Ceratonova shasta* | 50 - 65 | This study |
| CS_Ncol1-4-R | GCAACAAATTGGAGCAGGC |  |  |  |
| ML_Ncol1_4-F | TAAAATTCAAAGGACTTATC | *Myxidium lieberkuehni* | 50 - 65 | This study |
| ML_Ncol1_4-R | GGACATCCTYTAATRCATGT |  |  |  |
| SM_Ncol1_4-F | AGATTGCAATAGCTTTCAGC | *Sphaerospora molnari* | 50 - 65 | This study |
| SM_Ncol1_4-R | TTTCACCAGCCTAGTTCTTG |  |  |  |
